# Effects of Management Tactics on Meeting Conservation Objectives for Western North American Groundfish Fisheries

**DOI:** 10.1371/journal.pone.0056684

**Published:** 2013-02-27

**Authors:** Michael C. Melnychuk, Jeannette A. Banobi, Ray Hilborn

**Affiliations:** University of Washington, School of Aquatic and Fishery Sciences, Seattle, Washington, United States of America; Institute of Marine Research, Norway

## Abstract

There is considerable variability in the status of fish populations around the world and a poor understanding of how specific management characteristics affect populations. Overfishing is a major problem in many fisheries, but in some regions the recent tendency has been to exploit stocks at levels *below* their maximum sustainable yield. In Western North American groundfish fisheries, the status of individual stocks and management systems among regions are highly variable. In this paper, we show the current status of groundfish stocks from Alaska, British Columbia, and the U.S. West Coast, and quantify the influence on stock status of six management tactics often hypothesized to affect groundfish. These tactics are: the use of harvest control rules with estimated biological reference points; seasonal closures; marine reserves; bycatch constraints; individual quotas (i.e., ‘catch shares’); and gear type. Despite the high commercial value of many groundfish and consequent incentives for maintaining stocks at their most productive levels, most stocks were managed extremely conservatively, with current exploitation rates at only 40% of management targets and biomass 33% above target biomass on average. Catches rarely exceeded TACs but on occasion were far below TACs (mean catch**:**TAC ratio of 57%); approximately $150 million of potential landed value was foregone annually by underutilizing TACs. The use of individual quotas, marine reserves, and harvest control rules with estimated limit reference points had little overall effect on stock status. More valuable fisheries were maintained closer to management targets and were less variable over time than stocks with lower catches or ex-vessel prices. Together these results suggest there is no single effective management measure for meeting conservation objectives; if scientifically established quotas are set and enforced, a variety of means can be used to ensure that exploitation rates and biomass levels are near to or more conservative than management targets.

## Introduction

Marine fish populations around the world show a tremendous diversity of exploitation status. Some populations are severely overfished while others appear to be managed sustainably, with strong differences emerging at the regional level [1]. This regional variation likely involves differences in historical and current fishery management regimes, but it is unclear what particular aspects of management systems tend to lead to successful biological outcomes for some populations and overfishing for others. We can turn to regions in which stocks tend to be managed sustainably and ask what specific characteristics of those management systems tend to make them successful [2], [3].

Groundfish are fish species associated with the seafloor during their adult lives. Groundfish stocks, including rockfish, flatfish and roundfish such as cod, hake, and pollock, support important commercial fisheries around the world. The groundfish fisheries of western North America have a diverse history of exploitation. No Alaskan groundfish stocks have been overfished since the exclusion of foreign fleets in the late 1970s [1], [4]. In contrast, eight groundfish stocks off the U.S. West Coast (USWC) of Washington, Oregon and California have been classified as overfished [5]–[7]. In British Columbia, Canada (B.C.), four rockfish species have been designated as ‘Threatened’ by scientists (www.cosewic.gc.ca), but no recovery plans have been implemented at the political level (www.sararegistry.gc.ca). There is some debate regarding the appropriateness of IUCN population decline criteria (which are used by COSEWIC) to determine stock status for fisheries management [8], [9]. However, regardless of differences among regions in the specific criteria used to characterize population depletions, these criteria all form the basis for deciding whether to implement rebuilding plans for depleted stocks, and hence whether the targeting of a stock is reduced or prohibited. In addition to differences among regions, there is considerable diversity in the current status of individual groundfish stocks within each of these regions.

Fishery management systems are also diverse among these regions. For several decades Alaska has conducted extensive stock assessments and used harvest control rules for most stocks to limit overexploitation [4], [10] (harvest control rules are explicit rules for adjusting catches in response to observed changes in stock biomass). A wide range of methods are used to allocate quota to different fishing fleets and sectors. Stock assessments and harvest control rules were generally adopted somewhat later for the U.S. West Coast, after some of the overfished stocks had already been depleted. Severe catch restrictions for overfished stocks have resulted in many of these beginning to rebuild [1], [11]. In British Columbia, a system using trip limits transitioned into one using individual transferable quotas beginning in the mid-1990s [12]. The current system operates across several fleets and allows for a full accounting of catch mortality including discard mortality and bycatch [13]–[15]. Precautionary harvest control rules are used in British Columbia when estimates of stock status are available, but several stocks have not recently been assessed, thereby limiting the widespread use of harvest control rules. Individual quota systems have in the last few years been adopted for increasingly more stocks in Alaska and the U.S. West Coast. All regions conduct comprehensive fishery-independent surveys. In the context of global fisheries, the management systems in these regions may generally be regarded as successful, but the considerable variability in the particular aspects of how these management systems operate has likely influenced the variability in the biological status of individual stocks.

The conservation status of fish stocks is often framed in terms of recent biomass and exploitation rate (the proportion of the stock caught each year) relative to the levels that would in the long term produce maximum sustainable yield (MSY). In addition to reconstructed time series of biomass (B) and exploitation rate (F), stock assessments typically generate estimates of (or estimates of proxies for) the biomass, B_MSY_, and fishing mortality rate, F_MSY_, that would produce MSY. The total allowable catch (TAC) for a given stock is typically set based on the current stock status relative to these MSY-based biological reference points. For a variety of reasons stocks are sometimes overfished, including uncertain or biased stock status estimates and unreported or illegal catches. In other cases, stocks are ‘underfished’, or exploited at levels below the most productive yield possible [16]–[18]. Fish stocks undergo abundance fluctuations due to biological reasons beyond the control of management actions so we can not expect stocks to produce MSY every year, but maintaining stocks near their MSY-based targets on average is often considered to be a good management strategy.

Several hypotheses have been proposed about specific measures deemed necessary, sufficient, or contributory to successful fisheries management. Proponents of marine reserves suggest they are necessary for the protection of some stocks from fishing mortality [19], [20]. Spatial and/or temporal closures are used for western North American groundfish stocks to limit the extent of fishing effort, though the proportion of a stock's area of distribution or proportion of the year closed to fishing varies considerably. It is commonly suggested that a key ingredient to fisheries management is setting appropriate science-based catch limits and properly enforcing them [21]. Further, proponents of individual quota systems (i.e., ‘catch shares’) suggest that economic incentives for fishermen may promote resource stewardship and thus desirable conservation outcomes [22], [23]. Almost all western North American groundfish stocks are managed using annual TACs, though for some stocks these TACs are allocated to individuals while for others they are fished in an Olympic ‘race-to-fish’. The implementation of harvest control rules relies on stock assessment outputs, and these together are widely suggested as key measures for safeguarding against overexploitation [24], [25]. Assessments for most major groundfish stocks and several secondary target stocks are conducted in western North American regions, but the number of stocks within a region that are regularly assessed may limit the use of harvest control rules. Bycatch constraints are often adopted to limit the catch of sensitive or depleted fish stocks under rebuilding plans. In a multispecies fishery where several stocks are caught together, such bycatch limits may limit the quota allocated to more productive stocks in order to avoid further depleting the sensitive stocks; this certainly applies to western North American groundfish stocks [26]–[28].

We present an analysis of western North American groundfish stocks to quantify the influence on stock status of six management attributes often hypothesized to affect the status of groundfish. These attributes are: the use of harvest control rules with estimated biological reference points; the use of temporal closures; the extent of permanent or semi-permanent spatial closures; the level of catch constraints on target stocks from bycatch limits of sensitive species; the use of individual quotas; and the proportion of catch taken with bottom trawls. We consider as performance measures commonly-reported quantities representing stock status relative to management targets: the ratio of biomass to target biomass, the ratio of exploitation rate to target exploitation rate, the ratio of catch to TAC, as well as the proportion of catch discarded at sea. These performance measures have been increasingly used in recent years to compare the status of fish stocks [1], [29], [30], but few attempts have been made to attribute their variability to specific management measures. We recognize that predictor and response variables were not selected as part of a carefully controlled experiment and thus we are not able to demonstrate perfect causal relationships. However, since the predictor variables are generally employed independently of the response variables and cause-effect relationships between these variables are commonly hypothesized, we use terminology such as “influence of predictor variable on stock status” when describing the associations between variables.

## Results

### Current status of groundfish stocks

**Figure 1:**
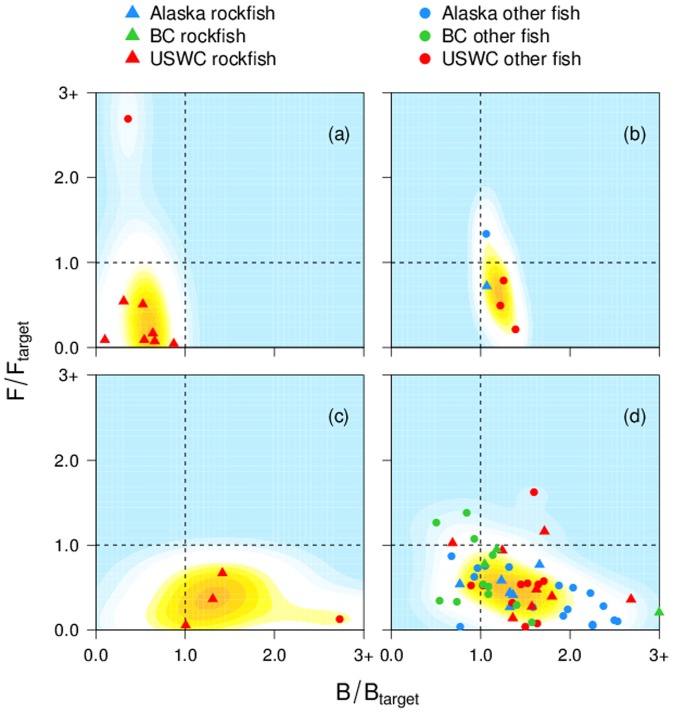
Current groundfish stock status in terms of recent exploitation rates to target exploitation rates and recent biomass to target biomass levels. Stocks are categorized as: (a) currently under a rebuilding plan; (b) previously under a rebuilding plan; (c) currently co-caught with rebuilding stocks; and (d) neither under a rebuilding plan nor co-caught with stocks under rebuilding. Stocks with little or no commercial value are excluded. Data points show averages of the most recent 5-year period available for each stock, and are separated by region and rockfish or other groundfish. Kernel density contour lines are shown, calculated over all data points assuming a bandwidth (smoothing parameter) of 2. Dashed lines show management targets for biomass and exploitation rate.

Groundfish stocks on the west coast of North America are, for the most part, managed very conservatively. These stocks include benthopelagic-oriented species such as walleye pollock, Pacific whiting, and spiny dogfish (see Supporting Information Table S1 for a full list of stocks). Stocks are generally defined separately for each of the three regions, with the exception of Pacific halibut and Pacific whiting, for which coastwide stock assessments are conducted. Over 70% of assessed groundfish stocks had recent biomass (5-year average) above the management target (Fig. 1); many of the depleted stocks, especially rockfish, are under formal rebuilding plans (Fig. 1a). Only 11% of targeted stocks had recent exploitation rates above the management target. The stock with the highest estimated exploitation rate in the last 5 years, USWC petrale sole, just entered into a rebuilding plan (Fig. 1a). Five stocks were previously under a rebuilding plan during their history, but recovered to near management targets for biomass and exploitation rate (Fig. 1b). A few stocks are currently co-caught with the USWC stocks under rebuilding plans (Fig. 1c), which limits exploitation rates of these co-caught stocks in order to reduce bycatch of rebuilding stocks. The majority of groundfish stocks have never been under a formal rebuilding plan, but are still fished conservatively, with exploitation rates of most stocks below levels (and biomass above levels) that are predicted to result in long term optimal yield (Fig. 1d).

Regional differences are apparent in terms of rebuilding plans for overfished stocks. No Alaskan groundfish stocks are currently or have recently been classified as overfished or under rebuilding plans. Several stocks from B.C. have been identified as possibly being ‘Threatened’ or of ‘Special Concern’, but no groundfish stocks are under recovery plans. Stocks from the USWC under rebuilding plans (Fig. 1a) or co-caught with rebuilding stocks (Fig. 1c) are not heavily targeted. Since we restrict the remainder of our analysis to targeted stocks, we exclude the stocks shown in Fig. 1a and c, and also exclude stocks with little or no commercial value.

### Diverse management tactics across regions

**Figure 2:**
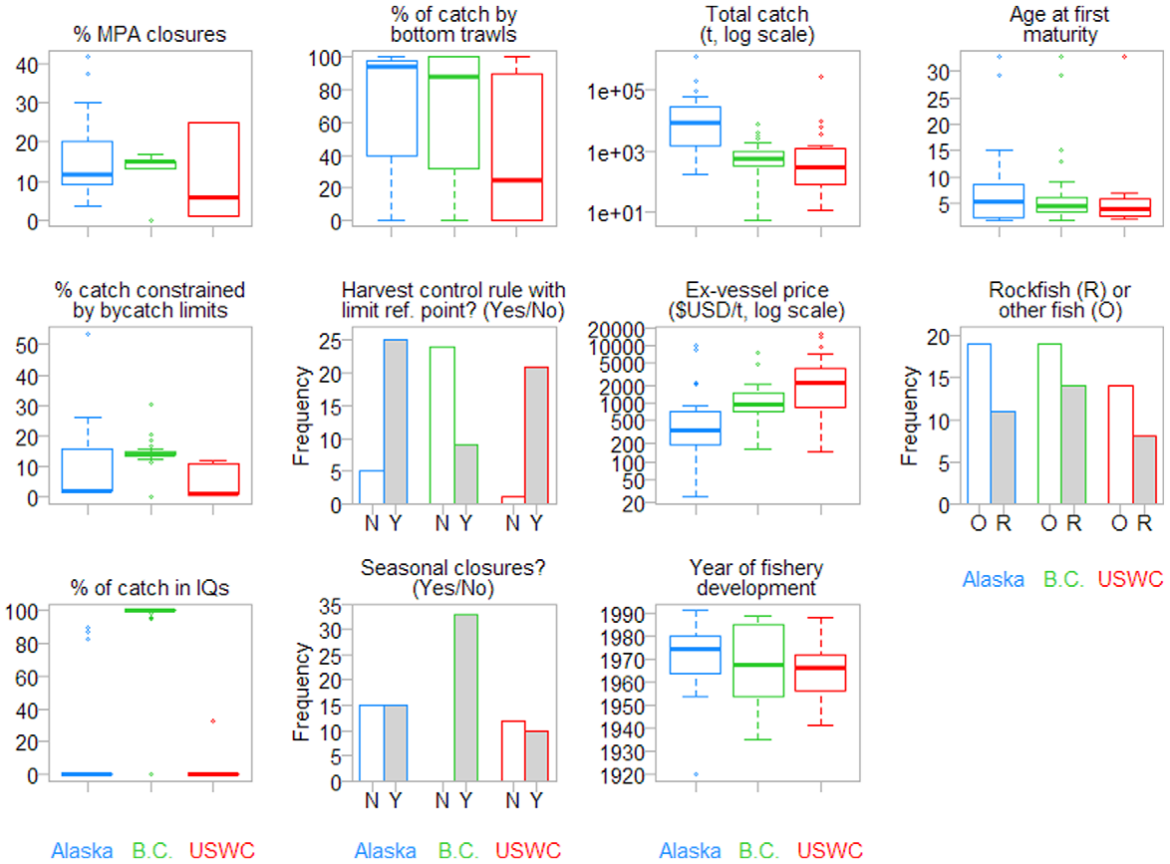
Boxplots of continuous predictor variables and barplots of categorical predictor variables used in analyses. Plots in two leftmost columns show management tactics and plots in two rightmost columns show other variables accounted for. Data are separated by region. See Table 3 for variable descriptions.

Tactics for managing groundfish fisheries are very diverse across stocks and regions (Fig. 2). The proportion of a stock's area under permanent spatial closures is similar for stocks from Alaska and B.C., typically 10–20% of the total area. Rockfish conservation areas on the USWC result in spatial closures of >20% for some stocks, but many stocks are unaffected by these closures so on average area closures are <10%. The estimated proportion of the hypothetical total catch that is constrained by bycatch limits (i.e., an estimate of foregone catch due to bycatch limits) typically ranged up to 15% for Alaskan and USWC stocks, but was >50% for Aleutian Islands walleye pollock, whose catch is constrained by protection measures for Steller sea lions. (Other forms of catch constraints, such as the 2 million t cap for Bering Sea groundfish, are not included in this measure.) This foregone catch was nearly 20% for most B.C. stocks, whose catch was constrained by bycatch limits for bocaccio and canary rockfish [31]. Strong regional differences were observed in the use of catch share programs. Nearly all groundfish stocks from B.C. had 100% of the total catch under individual transferable quotas. Although a few stocks from Alaska and the USWC were under partial catch share management including fishing co-operatives, most were competitive – still under limited entry systems, but without quota allocations to individuals (catch share programs have recently become more common in these regions, but the period of data availability is before these programs were implemented). The proportion of a stock's total catch caught by bottom trawls ranged widely from near 0% to 100%, but on average stocks from the USWC were more frequently caught with fixed gear – longlines or pots (59% of total catch by bottom trawl) – compared with stocks from Alaska (75%) and B.C. (69%). Harvest control rules with estimated limit biomass reference points are commonly employed for Alaskan and USWC stocks, but are employed for less than one third of B.C. stocks owing to a smaller proportion of stocks with assessments in which reference points are estimated. Finally, seasonal closures are used for essentially all groundfish stocks in B.C.; they are implemented mainly for lingcod and halibut spawning closures, but affect the fisheries for other stocks. In contrast, about 50% of the stocks from Alaska and the USWC were affected by seasonal closures.

We accounted for several other factors in our analyses that may influence groundfish stock status, and these also varied widely across stocks (Fig. 2). The median total catch of Alaskan stocks was about 10 times greater than that of B.C. or USWC stocks. Ex-vessel prices were typically lower for Alaskan stocks, and greatest for USWC stocks. Fisheries developed almost a decade later in Alaska, in the 1970s, than they did in B.C. or the USWC. The age at maturity was similar across the regions, as many species are found in two or all three of these regions, and because there was a similar proportion of rockfish stocks (compared to flatfish and roundfish) across regions. There is considerable variability among stocks in both management tactics (Fig. 2) and stock status (Fig. 1). While some stocks are larger than others in terms of biomass, catch, or landed value, all stocks were equally weighted, so are equally informative and influential for analyses. We now turn to look at the effects these management tactics have on biological performance measures describing stock status.

### Effects of management tactics on current stock status

**Figure 3:**
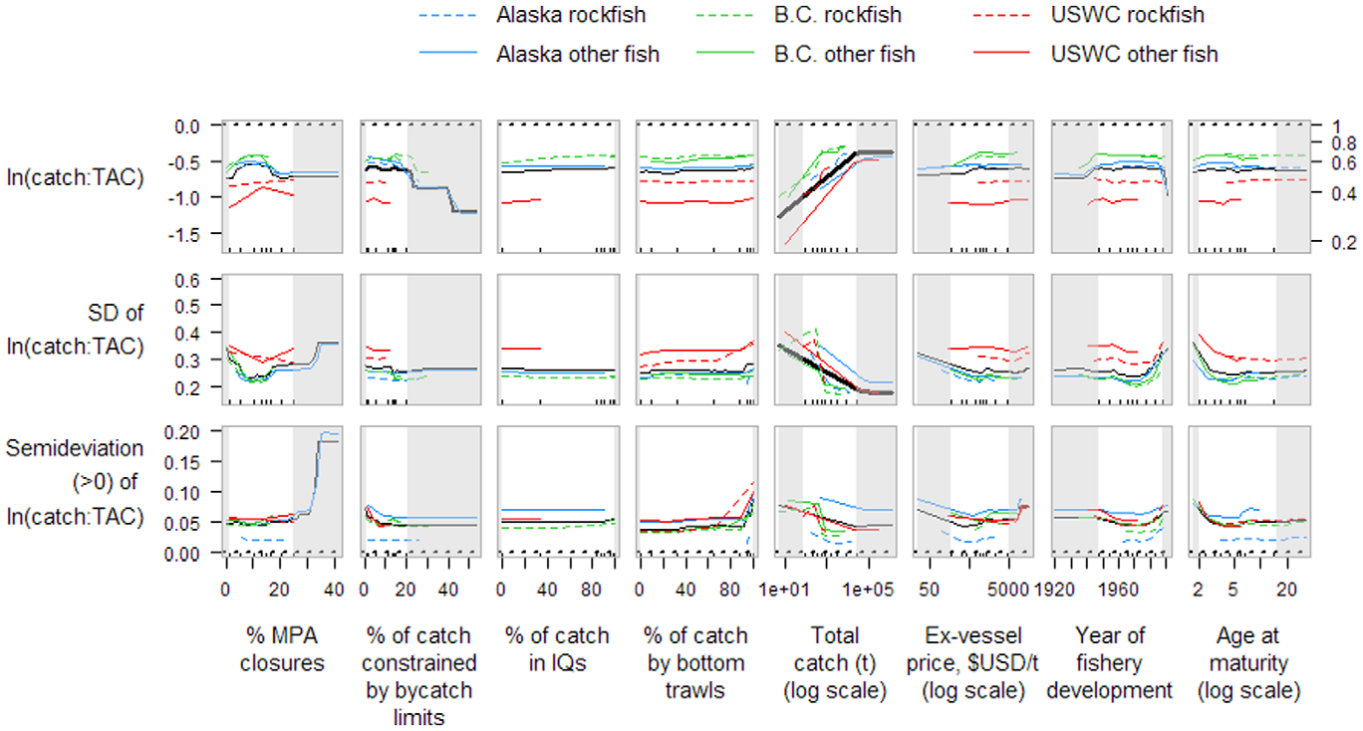
Partial dependence plots of three catch:TAC-related variables on eight numerical stock-level covariates of North American Pacific groundfish. The mean, standard deviation, and semideviation of the log-ratio of catch to TAC were calculated for each stock from the most recent 5-year period available. The three variables were analyzed independently using random forests (10,000 trees, 5 covariates randomly sampled at each split). The solid black line shows the marginal effect of a covariate across all stocks; thickness of the line is proportional to the covariate's relative importance score. Horizontal dotted lines at y = 0 represent general management objectives. Tick marks in each plot show deciles of covariate values; the region between grey-shaded areas contain 80% of the covariate values while grey-shaded areas contain the upper and lower 10% of covariate values. Right hand axis values show catch:TAC values on linear scale.

Total catches of western North American groundfish stocks were considerably less than annual TACs (mean catch**:**TAC ratio of 0.57; 5^th^, 50^th^ and 95^th^ percentiles of 0.16, 0.75, and 1.00). Stocks from B.C. tended to have recent catch**:**TAC ratios closest to the target (average, 0.69) than those from Alaska (0.57) or the USWC (0.39; Fig. 3). Stocks with seasonal closures had catch**:**TAC ratios that were considerably higher (closer to management targets) and less variable than stocks without seasonal closures (Table 1). Within the regions of greatest data density (i.e., focusing on the white regions in Fig. 3 instead of grey-shaded regions), we observed little influence on catch**:**TAC variables from the proportion of catch constrained by either spatial closures or bycatch limits, the proportion of catch under individual quota systems, or the proportion caught by bottom trawls. In contrast, we observed a strong effect of catch volume on the mean and variability of catch**:**TAC ratios. Stocks with greater total catch volume showed reduced variability in the ratio of catch**:**TAC as might be expected, but also had high catch**:**TAC ratios (closer to management targets), while smaller fisheries had ratios of catch**:**TAC much further from management targets (Fig. 3).

**Table 1:** Marginal mean responses of performance measures for each level of categorical management covariates.

Performance measure	Categorical covariate								
	HCR with limit RP?			Seasonal closures?			Taxa		
	No	Yes	% diff	No	Yes	% diff	Rockfish	non-RF	% diff
ln(catch**:**TAC)									
Mean	−0.63	−0.61	6.8%	−0.76	−0.57	61.3%	−0.63	−0.60	7.0%
Standard deviation	0.27	0.26	11.6%	0.28	0.25	25.1%	0.27	0.26	7.0%
Semi-deviation	0.051	0.051	0.2%	0.051	0.051	0.0%	0.052	0.050	4.3%
ln(F**:**F_target_)									
Mean	−0.90	−0.87	6.7%	−1.00	−0.87	27.4%	−0.94	−0.89	10.0%
Standard deviation	0.233	0.232	1.0%	0.235	0.231	4.6%	0.233	0.232	0.7%
Semi-deviation	0.076	0.060	24.1%	0.066	0.065	1.2%	0.064	0.067	5.4%
ln(B**:**B_target_)									
Mean	0.32	0.26	31.1%	0.31	0.27	17.9%	0.29	0.28	5.1%
Standard deviation	0.103	0.104	2.2%	0.098	0.105	13.1%	0.104	0.100	8.2%
Semi-deviation	0.110	0.095	16.4%	0.098	0.099	1.5%	0.098	0.099	0.1%
Discard proportion	15.1%	14.4%	8.6%	17.1%	13.7%	41.7%	15.2%	14.2%	11.7%

**Figure 4:**
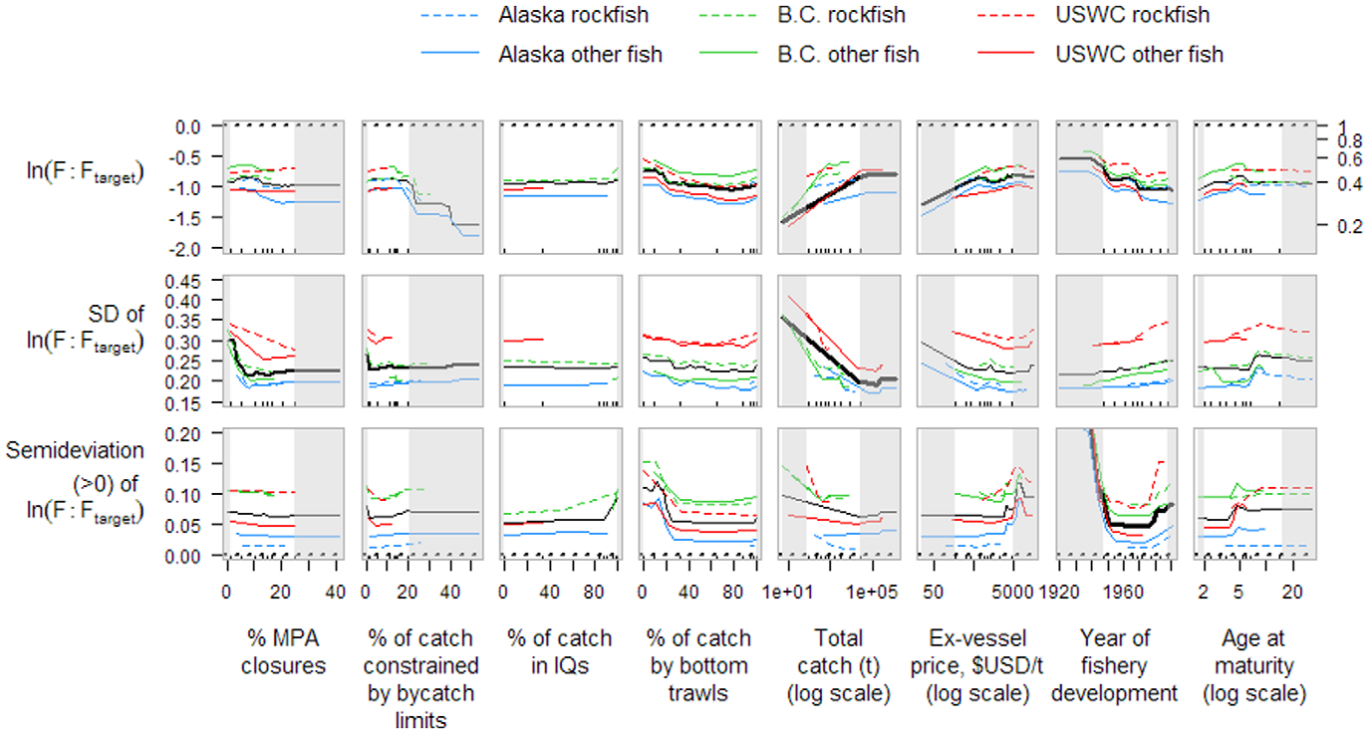
Partial dependence plots of three exploitation rate-related variables on eight numerical stock-level covariates of North American Pacific groundfish. The mean, standard deviation, and semideviation of the log-ratio of current exploitation rate to the target exploitation rate were calculated for each stock from the most recent 5-year period available. See Fig. 3 caption for further details.

**Figure 5:**
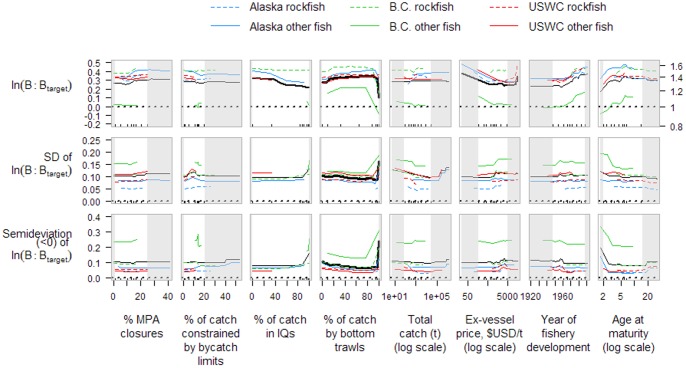
Partial dependence plots of three biomass-related variables on eight numerical stock-level covariates of North American Pacific groundfish. The mean, standard deviation, and semideviation of the log-ratio of current biomass to target biomass were calculated for each stock from the most recent 5-year period available. See Fig. 3 caption for further details.

**Figure 6:**
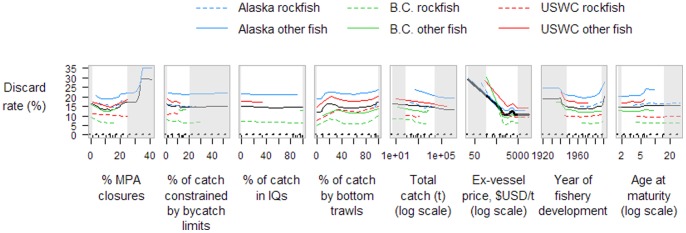
Partial dependence plots of discard proportion on eight numerical stock-level covariates of North American Pacific groundfish. Discard rates were calculated for each stock from the most recent 5-year period available. See Fig. 3 caption for further details.

Ten performance measures are shown, and for each random forest analysis (10,000 trees, 5 covariates randomly sampled at each split) the three categorical covariates were included along with the numerical covariates shown in Figs. 3–6. For each analysis, marginal means are given for both levels of each covariate. ‘% diff’ indicates the relative difference between the two levels, calculated as the absolute difference in means between the two levels as a percentage of the span of the 95% confidence interval of all data for the performance measure.

The mean ratio of current exploitation rate to target exploitation rates was only 0.40 (5^th^, 50^th^ and 95^th^ percentiles of 0.06, 0.52, and 1.27). Compared to the ratio of catch**:**TAC, there was less variation among regions in the mean ratio of F**:**F_target_ (Alaska, 0.34; B.C., 0.51; USWC, 0.41; Fig. 4). Stocks with seasonal closures had higher mean levels of F**:**F_target_, closer to management targets, compared to stocks without seasonal closures (Table 1). Groundfish stocks with areas of permanent spatial closures greater than 5–10% of the stock's distributional area had relatively low interannual variability in F**:**F_target_, but as %MPA (marine protected area) levels dropped below 5–10%, variability increased (Fig. 4). This was not, however, associated with a similar increase in the semi-deviation of F**:**F_target_ as %MPA levels decreased (semi-deviation is a measure of ‘downside risk’, or the asymmetrical variability of exceeding F_target_; see Methods), suggesting the high interannual variability observed did not involve exploitation rates exceeding management targets. Instead, stocks managed under harvest control rules which used limit reference points (implying also that stock assessments were conducted in which limit reference points were estimated) had lower semi-deviations of F**:**F_target_, while stocks managed without these harvest control rules and limit reference points had a greater tendency for exploitation rates to exceed target levels (Table 1). Although the interannual variability in F**:**F_target_ was similar across the entire range of the proportion of catch caught by bottom trawls, the semi-deviation increased as the % catch by bottom trawls dropped below about 20%, suggesting that stocks fished primarily with fixed gear or mid-water trawls had a greater tendency for exploitation rates to exceed F management targets (Fig. 4). Similar to the patterns observed for catch**:**TAC ratios, larger fisheries had low interannual variability and higher levels overall of F**:**F_target_, while smaller fisheries had higher variability and lower overall levels of F**:**F_target_, further away from management targets (Fig. 4). Stocks with relatively high ex-vessel prices (and also those that developed earlier) had higher F**:**F_target_ ratios, closer to management targets, than did lower-priced or later-developed stocks.

The mean ratio of current biomass to target biomass was 1.33 (5^th^, 50^th^ and 95^th^ percentiles of 0.68, 1.36, and 2.49). The biomasses of B.C. stocks were on average at target levels (1.04), while average biomasses of stocks from Alaska (1.45) and the USWC (1.42) were much higher (Fig. 5; with stocks under rebuilding plans included, the average biomass ratio for USWC stocks drops to 1.08). Flatfish and roundfish stocks from B.C. also showed greater interannual variability in B**:**B_target_ and, as average levels were so close to the management target, had a greater tendency for biomass levels to drop below target levels compared to stocks from other regions (Fig. 5). There was a slight tendency for B**:**B_target_ levels of Alaskan flatfish and roundfish stocks to be lower, closer to management targets, for stocks with a greater proportion of their catch under individual quotas. (There were insufficient data to evaluate biological responses after the establishment of recent Alaskan catch share programs —the Gulf of Alaska Rockfish Pilot Program and the Bering Sea Amendment 80 Program—so the only Alaskan stocks with IQs in this analysis were sablefish, halibut, and Bering Sea pollock.) Mean B**:**B_target_ levels were also lower, closer to management targets, for stocks that had harvest control rules with limit reference points (Table 1). Over most of the range of the proportion of total catch by bottom trawling, there was little observable effect of this covariate on any B**:**B_target_ variable. However, when total catches taken by bottom trawling were nearly 100%, the mean B**:**B_target_ was surprisingly much lower, closer to management target levels (Fig. 5). Interannual variation and especially the semi-deviation in B**:**B_target_ increased at this level of near-100% bottom trawling, suggesting that biomass had a greater tendency to fall below management targets for stocks caught exclusively by bottom trawling. Mean B**:**B_target_ levels were slightly lower, closer to target levels, for stocks with relatively early year of development and early year at maturity (Fig. 5).

Estimated discard rates ranged from 8.3% on average for B.C. rockfish stocks to 21.1% on average for Alaskan flatfish and roundfish stocks. Ex-vessel prices had the strongest influence by far on discard rates, with discard rates dropping from >20% at low prices to 10% at medium and high prices (Fig. 6). Effects of the other numerical covariates were negligible, although stocks with seasonal closures had lower discard rates on average than those without seasonal closures (Table 1).

### Exploratory and sensitivity analyses

We found limited reason for concern about the independence of categorical predictor variables (see Supporting Information Fig. S1). Similarly, we found little evidence of colinearity among the eight numerical predictor variables (all pairwise correlation coefficients were <0.5; Fig. S2). Scatterplots of observed response variables versus numerical predictor variables (Figs. S3, S4, S5) and scatterplots of observed versus predicted response variable values (Fig. S6) are also shown in the Supporting Information section. Partial dependence plots similar to Figs. 3, Fig. 4, Fig. 5,Fig. 6 are shown in the Supporting Information section for the five sensitivity analyses conducted (Fig. S7, Fig S8, Fig S9, Fig S10, Fig S11,Fig S12, Fig S13, Fig S14, Fig S15, Fig S16). In general, there were few noteworthy changes in the observed results from excluding stocks that are predominantly caught in recreational fisheries, filtering out less valuable stocks from the dataset (secondary target stocks or stocks with catch**:**TAC ratios <50%), or adding an extra predictor variable into random forest analyses (either region or maximum body length).

## Discussion

Conservation objectives of western North American groundfish fisheries are clearly being met for the vast majority of stocks, with catches, exploitation rates, and biomass levels adhering to management targets. There are few stocks whose recent catches exceed TACs or whose recent exploitation rates exceed targets. Of the stocks with recent biomass below target levels, most have had exploitation rates reduced to below MSY-based target levels and thus are expected to rebuild. Although overfishing is a problem in many regions of the world [1], [32], [33], in these three regions the general pattern across stocks appears to be quite different in recent years. Catches and exploitation rates are on average far below the levels that would in the long term provide maximum sustainable yield, greatest revenue, and maximum benefits of food security.

**Figure 7:**
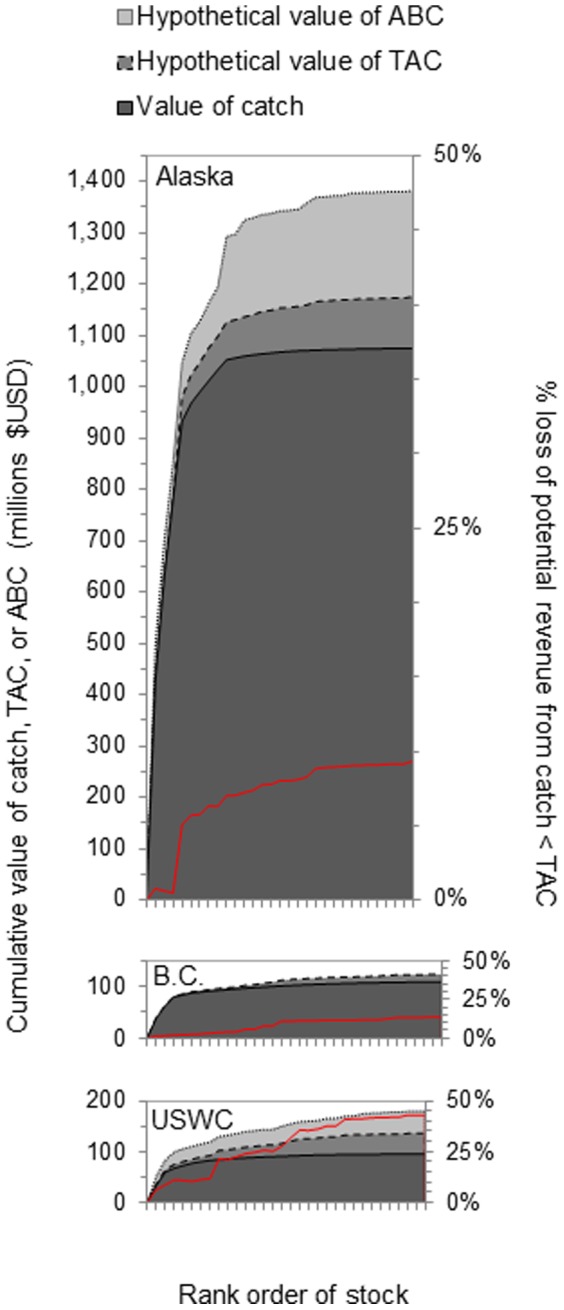
Cumulative distribution of groundfish value in Alaska, British Columbia, and the U.S. West Coast. The ex-vessel price of each stock is multiplied by the stock's total catch, total allowable catch (TAC), or allowable biological catch (ABC). Values represent recent 5-year means, and stocks are ranked within each region by catch value. % loss of potential revenue is calculated from the cumulative TAC–catch discrepancy as cumulative TAC/cumulative catch – 100%.

Overexploitation and underexploitation are both associated with loss of potential yield and revenue from a fished stock [16], [17]. If catch targets (TACs) are set adhering to sustainable yield recommendations from stock assessments, the loss of potential revenue from catching less than the TAC may be substantial. Based on simple calculations of stock-specific discrepancies between recent TAC and catch multiplied by ex-vessel prices (i.e., not accounting for long-term population dynamics impacts from set harvest policies), the total lost revenue from catching less than TAC in recent years was approximately 9% in Alaska (USD$ 100.7 million), 13% in B.C. ($14.7 million), and 43% on the U.S. West Coast ($40.8 million; Fig. 7). Catches generally adhere closely to TACs for the most valuable stocks in each region, but the cumulative percent loss of potential revenue increases as more of the lower value stocks are accounted for (Fig. 7). Allowable biological catches (ABC) are routinely estimated in assessments from Alaska and the USWC, and there is further revenue foregone from setting TACs lower than these ABC's (Fig. 7; TAC's are set lower than ABC's for a wide variety of ecological, social, and economic reasons). For some stocks, typically those of lower value, catches are below TACs due to market limitations. For others, even those with high ex-vessel prices, catches are below TACs because bycatch limits for weaker stocks are encountered before TACs for more targeted stocks are fulfilled [18]. This is especially the case for USWC stocks, where the percent loss of potential revenue for even the 10 most valuable stocks is >20% because TACs are not fully utilized (Fig. 7). Throughout the history of the USWC groundfish fishery, the loss in potential yield due to underexploitation (15–33% since 1990) has been considerably greater than the loss due to overexploitation (up to 3%) [18].

The total catch volume and ex-vessel price of stocks, both contributing to total value, were often stronger determinants of stock status than were any of the management tactics considered. The value of these groundfish fisheries was highly skewed towards a small number of species: in all three regions, the five most valuable stocks contributed 80–90% of the total catch value across all stocks (Fig. 7). More valuable stocks had lower discard rates and were kept closer to TAC, exploitation rate, and biomass targets than were less valuable stocks that were typically ‘underfished’. Similarly, fisheries that developed earlier were closer to exploitation rate and biomass targets than were later-developing fisheries. These findings are consistent with results from a global analysis at the species level, as the best business opportunities (higher prices, greater catch potential) are often developed first [34]. For more valuable fisheries, there is greater economic incentive to maintain catches closer to MSY levels each year. The interannual variability and tendency to exceed TAC and exploitation rate targets were consequently greater for stocks with lower average catch volume. This has important implications from a food security perspective. Stocks providing the greatest contributions towards food security (via magnitude of catch volume) are also the ones with a more reliable provision of food (via lower interannual variability), not by top-down design but rather because they're more valuable. This observed pattern for groundfish is unlikely to hold across all taxa. Small pelagic fish stocks, for example, often provide large catch volumes but are also highly variable interannually.

**Figure 8:**
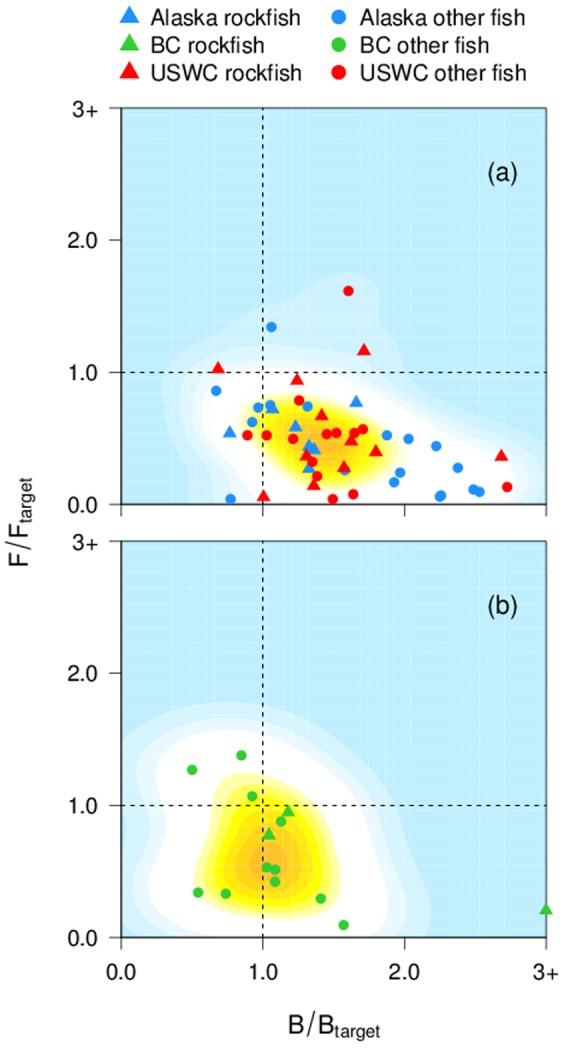
Influence of region and strength of adherence to harvest control rules on current groundfish stock status. Stock status is shown in terms of recent exploitation rates to target exploitation rates and recent biomass to target biomass levels. Stocks are categorized by the strength of adherence to harvest control rules: (a) automatic adherence; (b) discretionary adherence. Stocks with little or no commercial value, currently under a rebuilding plan, or co-caught with other stocks under rebuilding plans are excluded. See Fig. 1 caption for further details.

A harvest control rule which relies on estimated biological reference points is a management strategy that, if properly adhered to, should prevent overexploitation in the long term regardless of which management tactics are utilized to limit exploitation. Such rules [24], [35] are a transparent framework for adjusting exploitation rates in response to biomass changes. Stocks managed under harvest control rules with lower limit reference points had less tendency for exploitation rates and biomass to exceed management targets despite mean exploitation rate and biomass levels being closer to biomass targets. In other words, harvest control rules maintained stocks closer to management targets without exceeding them. This observation is confounded with regional differences, however, as assessments with estimated limit reference points are more common for the two U.S. regions (Fig. 2). On the USWC, several stocks were previously overfished, but after strong measures to cut exploitation rates were implemented, stocks have now rebuilt or are recovering [1], [11], [36]. Adherence to control rules is also stronger in the two U.S. regions (Fig. 8). Written into fisheries legislation, the targeting of a stock is automatically ceased if estimated biomass falls below the lower limit [24]. Conversely, in B.C. the response of managers and the federal fisheries Minister to decreases in estimated abundance is more discretionary. In B.C., managers and the fisheries Minister thus have more flexibility to also consider social and economic factors when deciding on management actions to rebuild depleted stocks. The observed effect of harvest control rules is therefore unclear in this study, as other management differences between B.C. and the two U.S. regions may confound effects on stock status.

Several management tactics for limiting the catch of fish stocks are employed around the world, consisting of output controls (e.g. individual quotas, trip limits, fleet-wide TAC caps), input controls (e.g. effort limits, gear restrictions, time and area closures), or a combination of these [1], [37]–[39]. We evaluated three of the most common tactics for constraining catch: temporal closures, permanent spatial closures, and bycatch limits of stocks co-caught with target stocks. In some ways, effects of these tactics on stock status may be moot as exploitation rates rarely exceeded management targets, so they are perhaps less useful as a catch-constraining tool compared to regions where stocks are more commonly overexploited. Still, some unexpected patterns emerged. Stocks with low proportions of spatial closures had increased variance in F**:**F_target_, but exploitation rates rarely exceeded targets even at these low levels of spatial closure. Stocks with seasonal closures had lower discard rates and considerably higher catch**:**TAC and F**:**F_target_ ratios, closer to management targets, but these are not necessarily causal relationships. Use of seasonal closures may be more likely for more valuable stocks in order to protect them during sensitive times like spawning periods, and these stocks may be maintained closer to their most productive levels and discarded less frequently because of their high value.

Bottom trawling has received considerable criticism in recent decades, not only for destructive habitat effects but also for the non-selective nature of the fishing gear [40]. Multispecies bottom trawl fisheries are generally thought to be less selective than mid-water trawls, pot, or longline fisheries [41], and bycatch limitations may thus be severe [26], [27]. For example, stocks from the USWC multispecies fisheries had the lowest catch**:**TAC ratios (furthest from management targets) and the greatest interannual variability in catch**:**TAC, largely as a result of such bycatch limits. Interestingly, the tendency to exceed management targets across all regions occurred at very low and high levels of bottom trawling, but not at intermediate levels. High semi-deviation of F**:**F_target_ occurred for stocks whose proportion of the total catch by bottom trawling was <20% and high semi-deviation of B**:**B_target_ occurred for stocks with >90% catch by bottom trawling (most apparent for stocks from B.C.). In the latter case, the frequency of falling below biomass targets was relatively high because biomass levels of stocks caught entirely by bottom trawls were lower, *closer* to management targets on average, than were biomass levels of stocks caught with a mixture of gear types or without bottom trawling. This suggests that bottom trawl fisheries can target individual stocks [28] well enough that on average stock biomasses are maintained near target levels even in multispecies fisheries.

Individual quota management has garnered much attention recently as a possible approach to end the race-to-fish. Catch-share systems are thought to provide incentives for fishermen to align their behavior with conservation objectives [22], [42], [43], and they have been shown to allow fisheries to better adhere to management targets and reduce interannual variation in catches and exploitation rates [30], [44]. Most groundfish stocks from B.C. are under full catch share management, but under the 5-year period of data availability only Pacific whiting from the USWC and Pacific halibut, sablefish, and walleye pollock from Alaska were under partial catch share systems. These are three of the four most valuable Alaskan stocks, and the economic incentive to maintain stocks near their most productive levels likely explains why biomass levels were lower, closer to management targets, for these stocks under catch shares. The non-catch share stocks, i.e. those managed with fleet-wide TAC caps but without allocation of quota to individuals, were also generally maintained within management targets. Thus it appears that as long as established TACs adhere to scientific recommendations and are adequately enforced, there are several possible allocation approaches for meeting groundfish conservation objectives. As a caveat, it is likely that catch share effects on response variables were underestimated for B.C. stocks because of the lack of contrast, i.e., there were very few non-catch share stocks (see Fig. 2) to contribute to observed relationships with response variables. The importance of catch shares to meeting management targets is likely especially important for B.C. stocks given that there are fewer stock assessments, less comprehensive survey programs, and less stringent enforcement of management plans compared to stocks in the U.S. regions.

Rockfish may be particularly susceptible to overfishing because they are often long lived and have relatively low fecundity, as has been shown for the California Current ecosystem [5], [7]. Although we did not detect any strong differences in recent stock status between rockfish and other groundfish after accounting for other factors, we restricted our analysis to targeted stocks, so we did not include the stocks under rebuilding plans from the USWC, most of which are rockfish (Fig. 1a). Taxonomic effects may also arise through the age of maturity covariate, as rockfish tend to mature relatively late; stocks of younger age at maturity were on average associated with lower biomass, closer to management targets, and a greater tendency to fall below target biomass.

While it is common practice to evaluate exploitation rates and biomass relative to targets, there is generally less consideration given to their interannual variability even though effects of management strategies and tactics may have a greater impact on variances than on mean responses [45]. Interannual variability may be as great a concern as the current stock status for fishermen who must make a living every year including the poor years [46]. Further, it is often interannual variability in stock status that leads to ratcheting effects, with fleets building up during good years to capacities beyond what can be sustained in poorer years [47]. Identifying management actions and strategies that reduce the interannual variability in stock status may therefore be an important goal along with identifying actions that lead to favorable stock status on average.

We found that management of groundfish stocks is highly conservative in western North American regions; these three regions tend to be considered successfully managed in the context of global fisheries. Overfishing of individual stocks was rare, and across all stocks the tendency was to catch less than science-based TACs. However, the relatively low exploitation rates and high biomass in these regions are not representative of other regions around the world where overexploitation is a more serious problem [1], [48]. These commercial fisheries also have a more recent history and fewer fishermen involved compared to many other regions like Europe and eastern North America, and this has possibly allowed for a greater conservation-oriented focus than other regions where social considerations are more prominent. No single management attribute stood out as being critical for sustainable management in these relatively successful regions. In the two U.S. regions, this success is likely attributable to strong science programs of surveys and assessments, legal requirements for conservation, and adequate enforcement. In B.C., catch shares are likely important for meeting stock management targets, despite not detecting this in analyses, as discussed above. Further studies at the global scale would provide more contrast in stock status, and in regions more characterized by overexploitation the influence of particular tactics on stock status may be stronger.

## Methods

### Data collection

We take a ‘snapshot’ approach and consider the most recent 5-year period of available data for each stock. Data used to calculate biological status were drawn from stock assessments. Data describing management attributes were compiled by reviewing assessments, fishery management plans, government databases or reports, the peer-reviewed literature, and especially by interviewing fisheries scientists, managers, and representatives of industry associations familiar with particular stocks. Values for management attributes corresponded to the same 5-year period of data availability for biological status. If a stock underwent a major management change during the most recent 5-year period (e.g., establishment of individual quota systems), we used instead the 5-year period immediately preceding the change.

All assessed stocks and many unassessed stocks from the three focal regions were included in our compiled databases. Time series and target reference point estimates for biomass and exploitation rates are routinely published in assessments. These are publicly available from the RAM Legacy Stock Assessment Database (ramlegacy.marinebiodiversity.ca) [29]. Occasionally time series estimates were available but target reference points were not estimated or published. In these few cases (4 of 60 stocks), a Schaefer [49] surplus production model was fit to time series data of total catch and total biomass estimates in order to estimate MSY-based reference points. The estimated ratios of B**:**B_MSY_ and F**:**F_MSY_ from a Schaefer model are a reasonable approximation to estimated ratios from stock assessments which typically use age-structured models [1], [30].

Although many of the stocks from these regions are assessed annually, for others assessments are conducted less frequently. If time series estimates from assessments were only available for years prior to 2000, we did not include the stock in our analysis. Because we consider measures of biological status relative to management targets, we include in our analysis only targeted stocks. We therefore also exclude stocks that had little or no commercial value, were under a rebuilding plan during the 5-year period, or were co-caught with stocks under rebuilding plans (Table S2; we show the current status of these stocks, but exclude them from random forest data analyses). After these exclusions a total of 85 stocks were included in our data set, and for any given performance measure data were available for 60–77 stocks (Table 2).

**Table 2:** Sample sizes for types of biological performance measures used in analyses.

Region Taxa	Catch and TAC data	F and F_target_ estimates	B and B_target_ estimates	% Discard estimates	
Alaska, U.S.					
Rockfish	11	8	7	11	
Other groundfish	19	18	18	19	
U.S. West Coast (continental)					
Rockfish	7	8	8	4	
Other groundfish	9	13	13	10	
British Columbia, Canada (B.C.)					
Rockfish	12	3	3	14	
Other groundfish	18	11	11	10	
Total	76	61	60	77	

Sampling units are individual groundfish stocks as defined in stock assessments. Stocks with little commercial value, under rebuilding plans in the last 5 years of data availability, or co-caught with stocks under rebuilding plans in the last 5 years are excluded.

### Biological status as performance measures

We considered performance measures that related to the biological status of groundfish stocks: biomass, exploitation rate, adherence of catch to TAC, and discard rate. For the first three of these, we explicitly accounted for management targets; performance measures consisted of the ratio of current biomass to target biomass, the ratio of current exploitation rate to target exploitation rate, and the ratio of current catch to current TAC. These targets are generally more conservative than values associated with maximum sustainable yield (e.g., B_40%_ is a conservative proxy for B_MSY_, the biomass level estimated to return long-term optimal yield). There is uncertainty as to which reference points are optimal [50], [51], and occasionally other targets are used by managers; we used as the denominator whatever was the explicitly stated management target. The ratio of catch**:**TAC represents a measure of implementation error [52] (more recently termed outcome error). To calculate this ratio, time series values were carefully screened to ensure that catches and TACs represented the same quantities in terms of spatial area, fishing gears, inclusion or exclusion of discards, and inclusion or exclusion of recreational catches. In some cases this involved using only a subset of the total catch of a stock so that it corresponded appropriately with the TAC value for the same year.

For each of these three types of performance measures, we calculated three metrics: the mean response over the 5-year period, the 5-year standard deviation around this mean, and the 5-year semi-deviation. Semi-deviation is often used as a measure of asymmetric risk around a target value: it represents the variability only on the undesirable side of the management target (i.e., catches exceeding TACs, exploitation rates exceeding target exploitation rate, and biomass below target biomass) [46], [53]. The 5-year semi-deviation around a target, δ(5, target), is zero if current values (*x_i_*) are at or more conservative than management targets throughout the 5-year period, and increases as current values become increasingly undesirable with respect to target values:
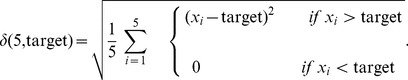
(1)
Equation 1 is expressed for the cases of 0 if catch*_i_* < TAC*_i_* or F*_i_* **<**F_target_; for biomass, the condition would instead switch to 0 if B*_i_* > B_target_. For all nine of these performance measures, the ratios were treated in log space. A tenth performance measure consisted of the proportion of a stock's total catch discarded at sea.

### Management tactics as predictor variables

We considered six management attributes that are commonly hypothesized to affect the biological status of fish stocks (Table 3):

**Table 3:** Fishery management covariates and other factors accounted for in analyses.

Covariate	Description
HCR with limit RP	Yes/no. Is there a harvest control rule used which involves a limit reference point? I.e., is there a stock size below which all directed fishing should stop? This implies that stock assessments are conducted in which limit reference points are estimated.
Seasonal closures	Yes/no. Are seasonal closures used?
% MPA closures	Numerical. The proportion of the stock's area of distribution under permanent spatial closure to fishing or extractive use (e.g., marine protected areas).
% catch constrained by bycatch limits	Numerical. The (hypothetical) proportion of the stock's total possible catch that is foregone as a result of bycatch limits for other exploited stocks or for threatened species (e.g., marine mammals).
% of catch in IQs	Numerical. The proportion of the total catch that is caught under catch share programs (e.g. individual quotas, co-operatives with allocated harvesting rights).
% of catch by bottom trawling	Numerical. The proportion of the total catch that is caught by bottom trawls.
Total catch	Numerical. Includes recreational catches and discards if data are available.
Ex-vessel price	Numerical. Average price for commercial landings.
Year of fishery development	Numerical. The year in which total landings first reached 25% of the historic maximum annual landings in the entire time series.
Age at maturity	Numerical. The average age at reproductive maturity.
Taxa	Boolean. Broad taxonomic division between rockfish and other groundfish, which include flatfish and roundfish.

All covariates were calculated or determined at the stock level for the same recent 5-year period considered for response variables.

- A harvest control rule is a management strategy for reducing exploitation rates in response to declines of stock biomass below a target reference point. Some harvest control rules require that fishing stops if estimated biomass falls below a lower limit reference point, which implies that this limit reference point is estimated in stock assessments. Stocks were classified by whether or not the latter type of harvest control rule was in place.
- Stocks were classified by whether or not seasonal closures were employed, which include either a period during the fishing season or the part of the year outside the fishing season. Closures may be designed for either the target stock or other stocks. We did not include cases where a fishery is closed for the remainder of a year simply after reaching the TAC.
- The proportion of a stock's distributional area that is under spatial closure to fishing or extractive use (e.g., marine reserves; rockfish conservation areas). If distributional areas were not available, the area over which fishing typically occurs was used instead as the denominator. If a spatial closure did not impact the gear(s) with which a stock is normally caught (e.g. mid-water trawls), it was not considered a closure for that stock. Rotational or temporary closures were not counted.
- The level of catch constraints due to bycatch limits for other species was estimated by fishery managers and/or industry association representatives. This is a subjective measure of the foregone catch of the target stock, expressed as a percentage of the hypothetical total catch in the absence of any bycatch limits. Bycatch limits may be for other exploited stocks, or for threatened species such as Steller sea lions in the Aleutian Islands. Note that four stocks were excluded from analyses because they were frequently co-caught with rebuilding stocks (Table S2), but catch constraints may also apply to the remaining 85 stocks.
- The proportion of a stock's total catch under catch share programs. These programs include any quota system that allocates harvesting rights to individuals, such as individual transferable, vessel, or fishing quotas (ITQ, IVQ, IFQ) and industry co-operatives with quota allocations.
- The proportion of a stock's total catch that was taken by bottom trawls.

Other covariates that may potentially influence performance measures were also considered (Table 3). The total catch and ex-vessel price contribute to the value of a fishery, and there may be greater attention given to more valuable fisheries to ensure that management objectives are met [34]. The year of fishery development was determined for each stock, defined as the first year in which the total landings reached 25% of the maximum historic landings in the full time series [34]. The average age at maturity represents an important life history trait affecting a stock's potential to rebuild. Values were drawn at the species level from FishBase. Finally, a taxonomic division was considered between rockfish and other groundfish, as the biological status and influence of management tactics may differ between these groups.

### Data analyses

We used random forests to assess the influence of management tactics and other covariates (i.e., predictor variables) on the biological performance indicators (response variables). Random forests [54] are an ensemble of regression trees, which are a non-parametric recursive data splitting method for identifying covariates with relatively strong influence on a numerical response variable. At a given node of a regression tree, values of the response variable and one predictor variable are split into two groups based on whichever predictor variable's split results in the greatest sum of squares reduction of the response variable. The procedure is repeated such that within a single tree, multiple predictor variables can be shown to influence the response variable. Although single regression trees are unstable in terms of the order of variable importance among covariates, random forests involve bootstrapping the dataset (each component regression tree is constructed from one resampled dataset) and only allowing a random subset of covariates to be included at any given node of a component tree, with the result being a more robust measure of variable importance across the aggregated set of trees [55]. Random forests have been used increasingly in ecology and fisheries research [46], [56], [57]. They are attractive for cases like ours in that they allow for non-linear relationships between a predictor and response variable, they do not make any parametric assumptions about the distribution of a response variable, interactions among predictor variables are accounted for implicitly, they can handle missing values of predictor variables, and they are less susceptible to over-fitting compared with parametric methods such as generalized linear models because the number of predictor variables available for selection at any given node of a tree is limited to a specified number.

For each of the ten performance measures, we conducted a separate random forest analysis using the ‘randomForest’ package (version 4.6-2) [58] in R (version 2.14.1) [59]. Stocks were weighted equally. Forests of 10,000 trees were used, which were more than adequate from visual inspection of model diagnostics. The cross-validation prediction accuracy represented by the mean square error of model fit is sensitive to *mtry*, a tuning parameter which limits the number of predictor variables allowed for selection at any one node of a component regression tree. Larger values of *mtry* are often less susceptible to overfitting large models and allow for higher order interactions among predictors, while smaller values of *mtry* often have greater cross-validation prediction accuracy. The mean square error of model fit was plotted over a range of *mtry* values to determine an appropriate value. A value of *mtry* = 5 was selected based on these diagnostics, which is reasonably close to rules of thumb of *mtry*≈ 1/3*p* for continuous predictors or √*p* for categorical predictors, where *p* is the number of predictor variables (in our case, *p* = 11).

We show effects of management tactics and other covariates on response variables using partial dependence plots for the eight continuous predictors and marginal means of performance measures for the three categorical predictors listed in Table 3. Partial dependence plots show the effect of a predictor variable of interest on a response variable after accounting for the average effects of the other predictor variables in the model. At a given value of predictor variable *x*, a value of the response variable is predicted from all the combinations of observed values of the other predictor variables in the random forest dataset, and the average predicted response variable is determined. This process is repeated for many values of *x* to construct a dependence plot (see [58] for further details). Partial dependence plots are not constrained by linear relationships through the range of covariate values. We show marginal relationships for six separate groups (3 regions × categories of rockfish or other groundfish) as well as the overall marginal relationship. Although ‘region’ was not included as a predictor variable in the main analysis (only in a sensitivity analysis), the three regions differ in their values of other predictor variables, which permits separate partial dependence functions to be calculated for each region. We show a measure of relative predictor variable importance for each of the ten random forest analyses. The importance score is determined with cross-validation and reflects the loss of prediction accuracy associated with removing each predictor variable in turn (for further details see the ‘importance {randomForest}’ function in [58]). As the modest sample sizes available sometimes resulted in sparse data in the tails of predictor variable values, we draw the reader's attention in these partial dependence plots to the middle 80% of the values of each predictor variable. For categorical predictor variables, we show the difference in marginal means of response variables between the different levels of the predictor. We express this difference as a proportion of the span of the 95% confidence interval of response variable values, which provides a relative index of the effect of categorical predictors on a response variable.

Prior to analyses, we tested for colinearity among predictor variables using generalized variance inflation factors (GVIF) [60]. All GVIF values were <2.5 suggesting little possibility of confounding among the 11 predictor variables [60]. (We had originally considered ‘region’ as a twelfth predictor, categorical with 3 levels, but this was highly confounded with other predictors on the basis of high GVIF scores so was dropped as a predictor.) We confirmed this visually, plotting all pair-wise combinations of the eight continuous predictor variables and finding little evidence of colinearity. Similarly, we inspected mosaic plots of pair-wise combinations of the three categorical predictors and found limited reason for concern about the independence of these predictors. We plotted response variables versus predictor variables to visually assess relationships and plotted observed versus predicted response variable values to visually assess model fit. Finally, we conducted five sensitivity tests for random forest analyses to assess the influence on observed results of subsetting the dataset or adding predictor variables into the models:

- We excluded stocks if >50% of their total catch was taken by the recreational sector.
- We excluded stocks if their catch**:**TAC ratio was <50%, as this likely represents stocks that are not heavily targeted.
- We excluded stocks identified by managers or in fishery management plans as being secondary targets. Note these exclusions a–c are in addition to the exclusions mentioned previously (stocks with little or no commercial value, under rebuilding plans, or co-caught with rebuilding stocks), which were common across all analyses.
- We added a 3-level ‘region’ predictor variable to the model even though it was highly confounded with other predictor variables in order to assess whether observed effects of other predictors would change once region was accounted for explicitly (rather than implicitly, through differences among regions in the ranges of covariate values).
- We added maximum length (drawn at the species level from FishBase) as a continuous predictor variable in the model to allow for a second life history variable to possibly explain variation in performance measures.

## Supporting Information

Figure S1 **Values of management-related categorical covariates used in the analysis.** Mosaic plots are shown for each of the three pair-wise combinations of categorical covariates.
(TIF)

Figure S2 **Values of management-related numerical covariates used in the analysis.** Lower panels show pair-wise scatterplots between covariates. Upper panels show correlation coefficients for the same pairs. Histograms of covariate values are shown on the diagonal. A Lowess fit with smoothing parameter = 2 is shown on each scatterplot. Data points show values for individual stocks, separated by color: Alaska—blue, U.S. west coast—red, B.C.—green; and by symbol: rockfish—triangles, other groundfish—circles.
(TIF)

Figure S3 **Scatterplots of three catch:TAC response variables versus eight numerical stock-level covariates.** The mean, standard deviation, and semideviation of the log-ratio of catch to TAC were calculated for each stock from the most recent 5-year period available. Data points are shown by region and rockfish/other groundfish groupings. Horizontal dotted lines at y = 0 represent general management objectives. Right hand axis values show catch:TAC values on linear scale.
(TIF)

Figure S4 **Scatterplots of three F:F_target_ response variables versus eight numerical stock-level covariates.** See Fig. 3 caption for details.
(TIF)

Figure S5 **Scatterplots of three B:B_target_ response variables versus eight numerical stock-level covariates.** See Fig. 3 caption for details.
(TIF)

Figure S6 **Scatterplots of observed versus predicted response variable values.** Three metrics (mean, standard deviation, and semideviation of the most recent 5-year period of data available) for each of three variables (catch:TAC, F:F_target_, and B:B_target_) were calculated for each stock. Predicted values are from the key run of random forest analyses. Dotted line shows the 1∶1 relationship.
(TIF)

Figure S7 **Partial dependence plots for sensitivity analyses showing the influence of numerical covariates on the mean catch:TAC ratio.** The key run and five sensitivity scenarios are labelled in the right margin. See Fig. 3 caption in main text for further details.
(TIF)

Figure S8 **Partial dependence plots for sensitivity analyses showing the influence of numerical covariates on the interannual variability of the catch:TAC ratio.** The key run and five sensitivity scenarios are labelled in the right margin. See Fig. 3 caption in main text for further details.
(TIF)

Figure S9 **Partial dependence plots for sensitivity analyses showing the influence of numerical covariates on the semi-deviation of the catch:TAC ratio.** The key run and five sensitivity scenarios are labelled in the right margin. See Fig. 3 caption in main text for further details.
(TIF)

Figure S10 **Partial dependence plots for sensitivity analyses showing the influence of numerical covariates on the mean F:F_target_ ratio.** The key run and five sensitivity scenarios are labelled in the right margin. See Fig. 3 caption in main text for further details.
(TIF)

Figure S11 **Partial dependence plots for sensitivity analyses showing the influence of numerical covariates on the interannual variability of the F:F_target_ ratio.** The key run and five sensitivity scenarios are labelled in the right margin. See Fig. 3 caption in main text for further details.
(TIF)

Figure S12 **Partial dependence plots for sensitivity analyses showing the influence of numerical covariates on the semi-deviation of the F:F_target_ ratio.** The key run and five sensitivity scenarios are labelled in the right margin. See Fig. 3 caption in main text for further details.
(TIF)

Figure S13 **Partial dependence plots for sensitivity analyses showing the influence of numerical covariates on the mean B:B_target_ ratio.** The key run and five sensitivity scenarios are labelled in the right margin. See Fig. 3 caption in main text for further details.
(TIF)

Figure S14 **Partial dependence plots for sensitivity analyses showing the influence of numerical covariates on the interannual variability of the B:B_target_ ratio.** The key run and five sensitivity scenarios are labelled in the right margin. See Fig. 3 caption in main text for further details.
(TIF)

Figure S15 **Partial dependence plots for sensitivity analyses showing the influence of numerical covariates on the semi-deviation of the B:B_target_ ratio.** The key run and five sensitivity scenarios are labelled in the right margin. See Fig. 3 caption in main text for further details.
(TIF)

Figure S16 **Partial dependence plots for sensitivity analyses showing the influence of numerical covariates on the proportion of catch discarded.** The key run and five sensitivity scenarios are labelled in the right margin. See Fig. 3 caption in main text for further details.
(TIF)

Table S1 **Stocks included in random forest data analyses (n = 85).**
(DOCX)

Table S2 **Stocks excluded from stock status presentation and from random forest data analyses.**
(DOCX)

Text S1 **This section contains the results of exploratory data analyses and sensitivity tests as mentioned in the main text.** Also listed are the groundfish stocks that were included in analyses (Table S1), and stocks excluded from analyses (Table S2).
(DOCX)
