# Correction: Effects of Management Tactics on Meeting Conservation Objectives for Western North American Groundfish Fisheries

**DOI:** 10.1371/journal.pone.0210843

**Published:** 2019-01-10

**Authors:** Michael C. Melnychuk, Jeannette A. Banobi, Ray Hilborn

There is additional information that needs to be included in the Competing Interests statement. The correct Competing Interests statement is as follows: Funding was received by RH in the five-year period prior to the publication of this article from sources including fishery/seafood corporations and industry groups, as well as environmental groups and foundations (please see the list attached here as Supporting Information S1 File). While this funding did not directly support the research reported in this article, the authors are declaring it in accordance with *PLOS ONE* policy. This does not alter our adherence to *PLOS ONE* policies on sharing data and materials.

The funding that directly supported the work reported in this article is already listed accurately and in full in the Funding statement.

## Supporting information

S1 File Funding received by RH during the 5 years prior to publication of this article. (XLSX)
