# Supplementary figures and images for: Effects of Management Tactics on Meeting Conservation Objectives for Western North American Groundfish Fisheries
Source: PLoS One. 2013 Feb 27;8(2):e56684. doi: 10.1371/journal.pone.0056684 (PMC3584066; doi:10.1371/journal.pone.0056684)

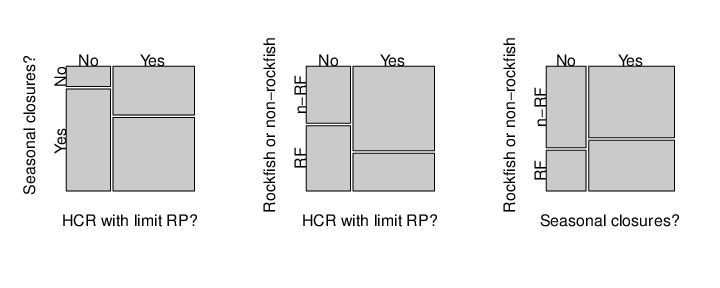

Supplement: Figure S1 — Values of management-related categorical covariates used in the analysis. Mosaic plots are shown for each of the three pair-wise combinations of categorical covariates. (TIF) [file pone.0056684.s001.tif]

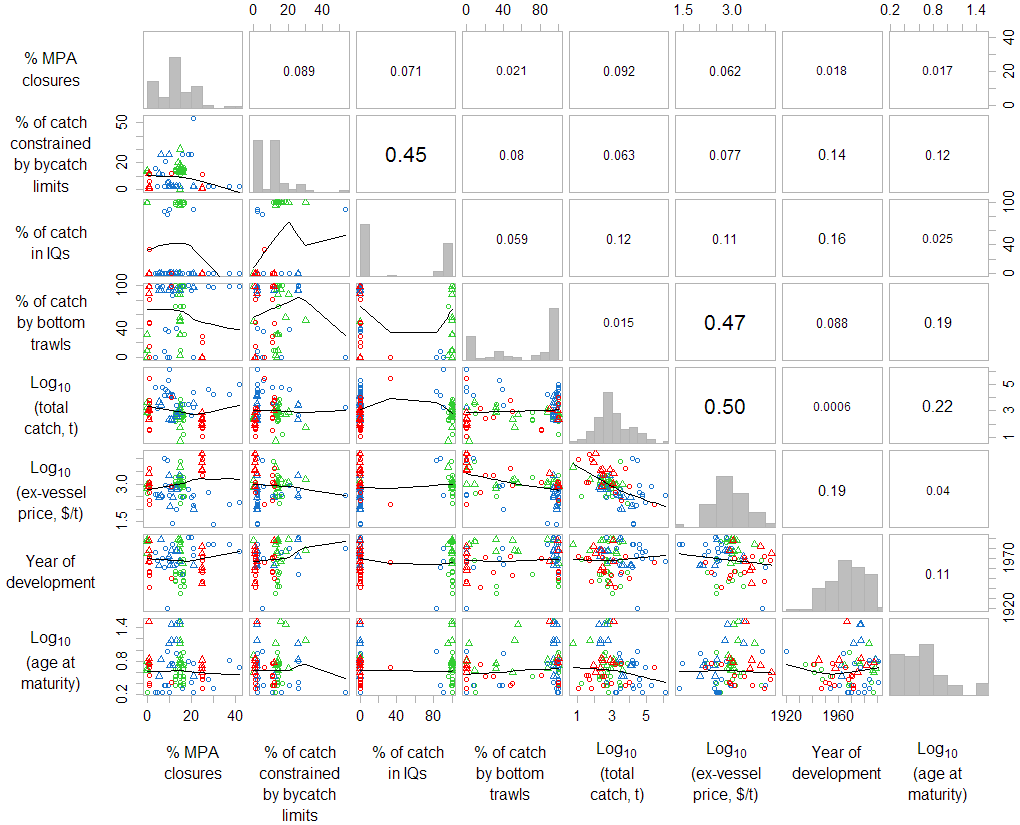

Supplement: Figure S2 — Values of management-related numerical covariates used in the analysis. Lower panels show pair-wise scatterplots between covariates. Upper panels show correlation coefficients for the same pairs. Histograms of covariate values are shown on the diagonal. A Lowess fit with smoothing parameter = 2 is shown on each scatterplot. Data points show values for individual stocks, separated by color: Alaska—blue, U.S. west coast—red, B.C.—green; and by symbol: rockfish—triangles, other groundfish—circles. (TIF) [file pone.0056684.s002.tif]

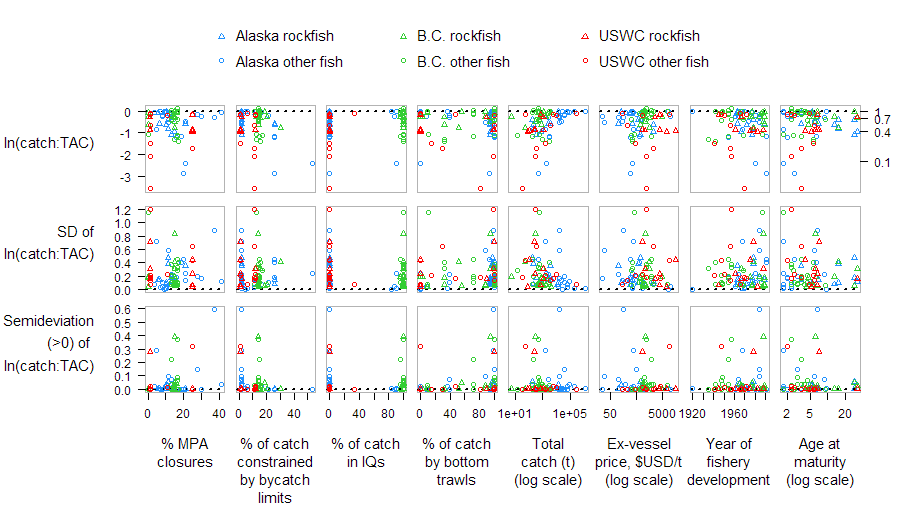

Supplement: Figure S3 — Scatterplots of three catch:TAC response variables versus eight numerical stock-level covariates. The mean, standard deviation, and semideviation of the log-ratio of catch to TAC were calculated for each stock from the most recent 5-year period available. Data points are shown by region and rockfish/other groundfish groupings. Horizontal dotted lines at y = 0 represent general management objectives. Right hand axis values show catch:TAC values on linear scale. (TIF) [file pone.0056684.s003.tif]

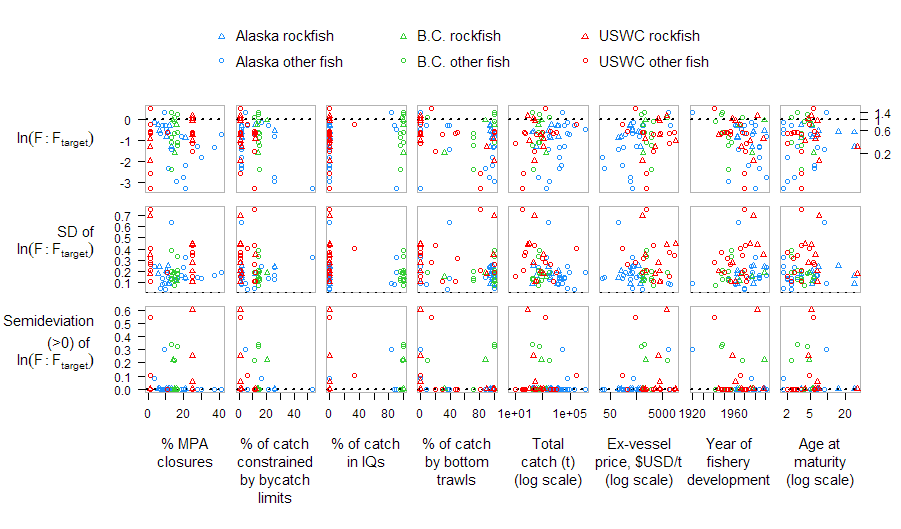

Supplement: Figure S4 — Scatterplots of three F:Ftarget response variables versus eight numerical stock-level covariates. See Fig. 3 caption for details. (TIF) [file pone.0056684.s004.tif]

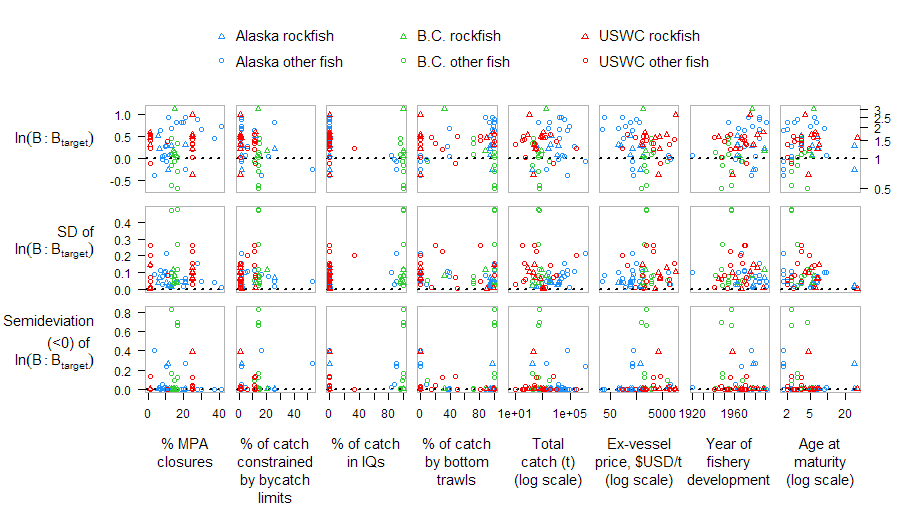

Supplement: Figure S5 — Scatterplots of three B:Btarget response variables versus eight numerical stock-level covariates. See Fig. 3 caption for details. (TIF) [file pone.0056684.s005.tif]

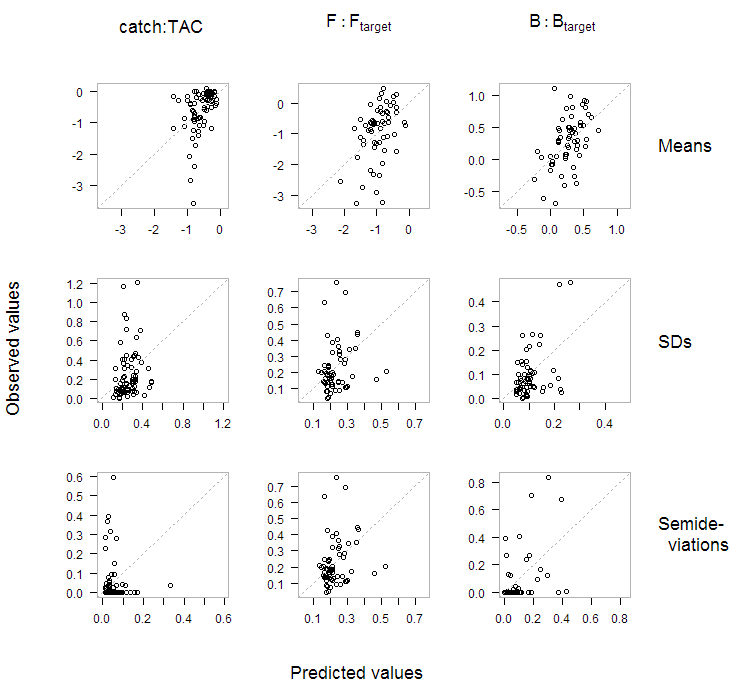

Supplement: Figure S6 — Scatterplots of observed versus predicted response variable values. Three metrics (mean, standard deviation, and semideviation of the most recent 5-year period of data available) for each of three variables (catch:TAC, F:Ftarget, and B:Btarget) were calculated for each stock. Predicted values are from the key run of random forest analyses. Dotted line shows the 1∶1 relationship. (TIF) [file pone.0056684.s006.tif]

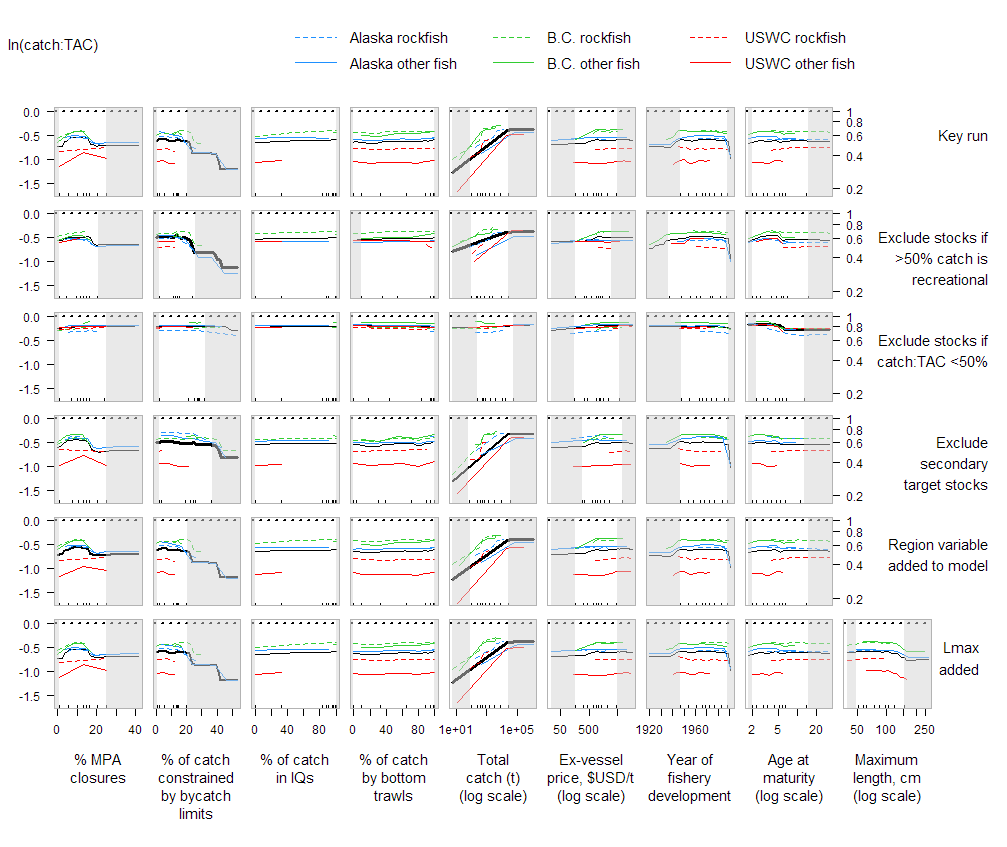

Supplement: Figure S7 — Partial dependence plots for sensitivity analyses showing the influence of numerical covariates on the mean catch:TAC ratio. The key run and five sensitivity scenarios are labelled in the right margin. See Fig. 3 caption in main text for further details. (TIF) [file pone.0056684.s007.tif]

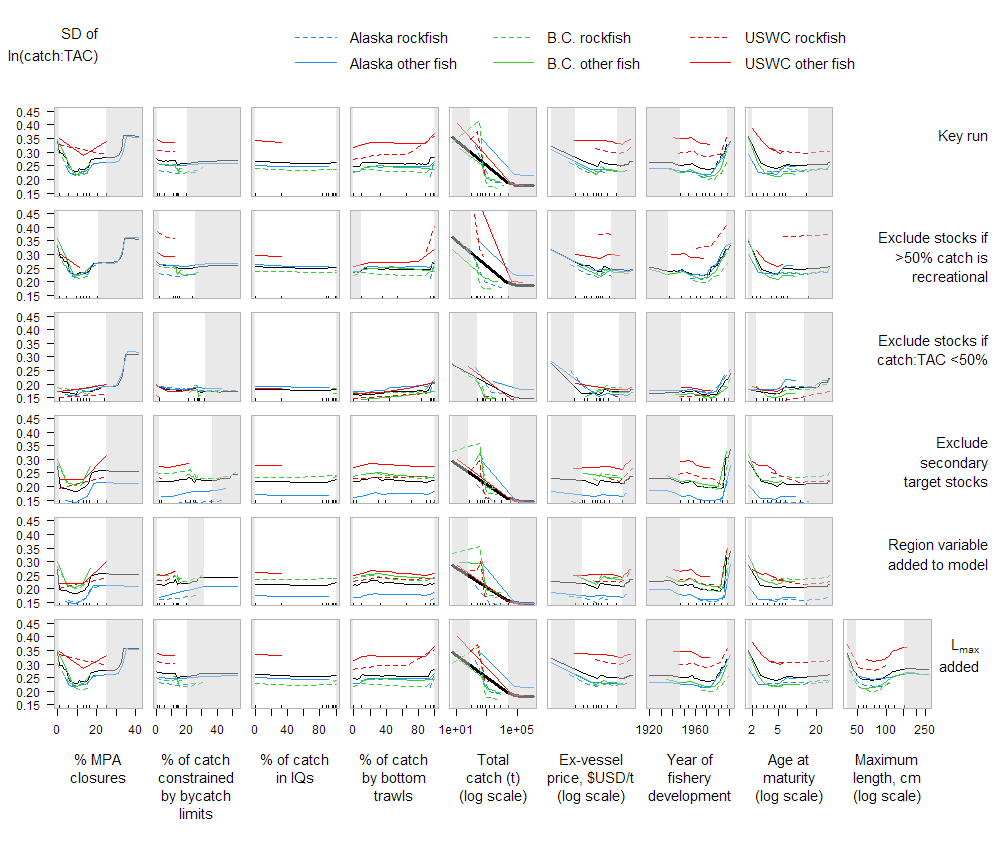

Supplement: Figure S8 — Partial dependence plots for sensitivity analyses showing the influence of numerical covariates on the interannual variability of the catch:TAC ratio. The key run and five sensitivity scenarios are labelled in the right margin. See Fig. 3 caption in main text for further details. (TIF) [file pone.0056684.s008.tif]

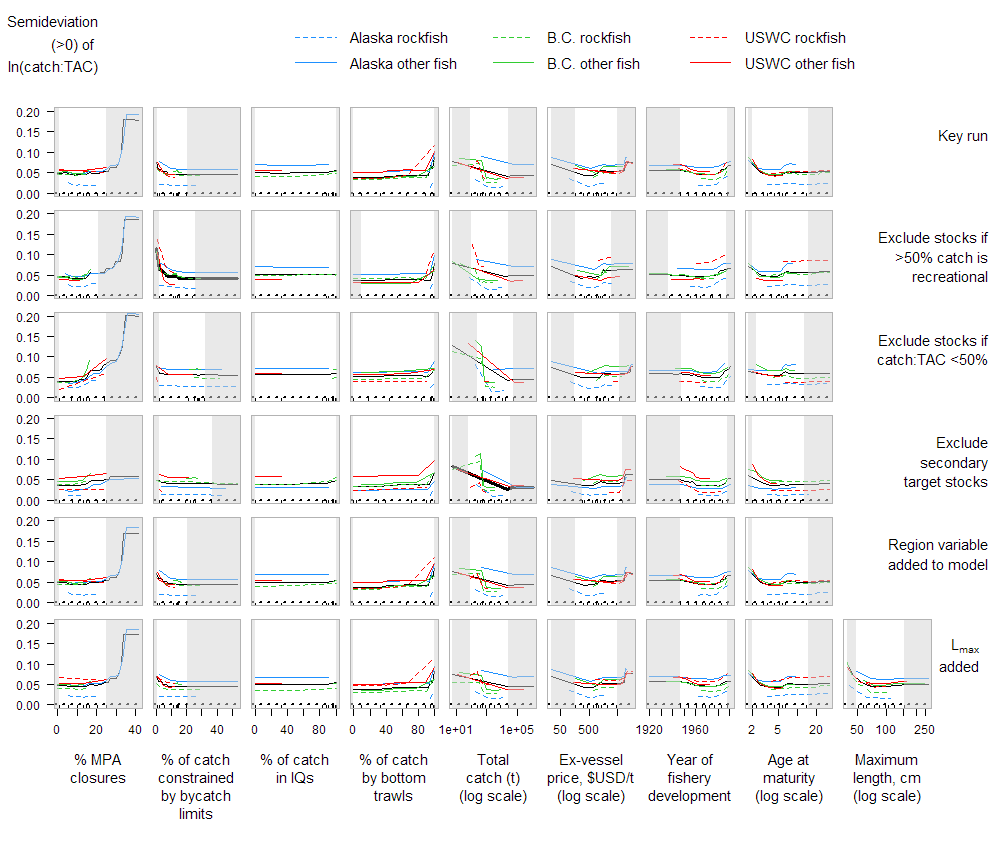

Supplement: Figure S9 — Partial dependence plots for sensitivity analyses showing the influence of numerical covariates on the semi-deviation of the catch:TAC ratio. The key run and five sensitivity scenarios are labelled in the right margin. See Fig. 3 caption in main text for further details. (TIF) [file pone.0056684.s009.tif]

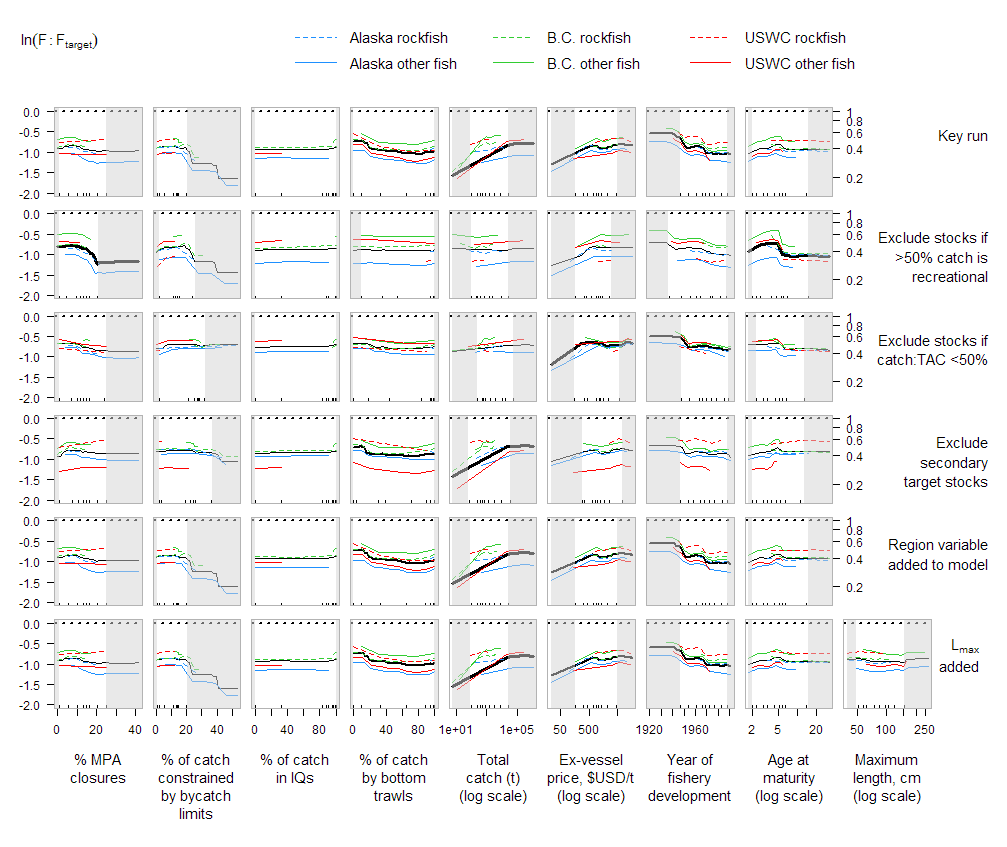

Supplement: Figure S10 — Partial dependence plots for sensitivity analyses showing the influence of numerical covariates on the mean F:Ftarget ratio. The key run and five sensitivity scenarios are labelled in the right margin. See Fig. 3 caption in main text for further details. (TIF) [file pone.0056684.s010.tif]

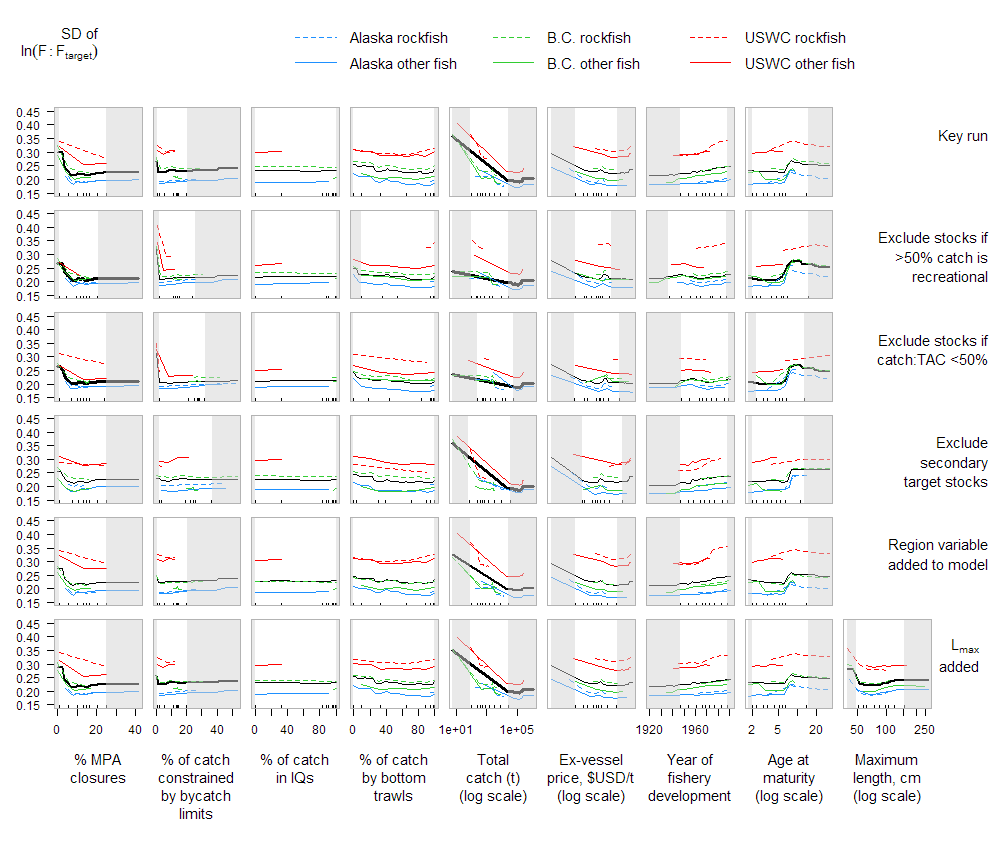

Supplement: Figure S11 — Partial dependence plots for sensitivity analyses showing the influence of numerical covariates on the interannual variability of the F:Ftarget ratio. The key run and five sensitivity scenarios are labelled in the right margin. See Fig. 3 caption in main text for further details. (TIF) [file pone.0056684.s011.tif]

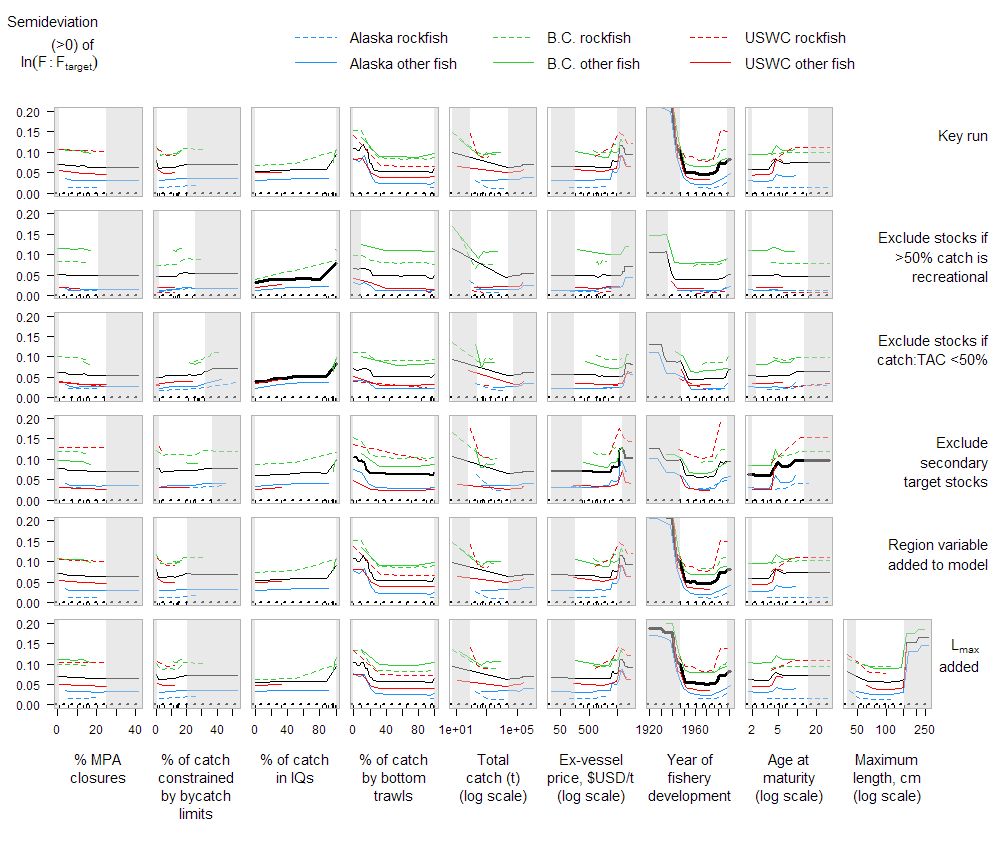

Supplement: Figure S12 — Partial dependence plots for sensitivity analyses showing the influence of numerical covariates on the semi-deviation of the F:Ftarget ratio. The key run and five sensitivity scenarios are labelled in the right margin. See Fig. 3 caption in main text for further details. (TIF) [file pone.0056684.s012.tif]

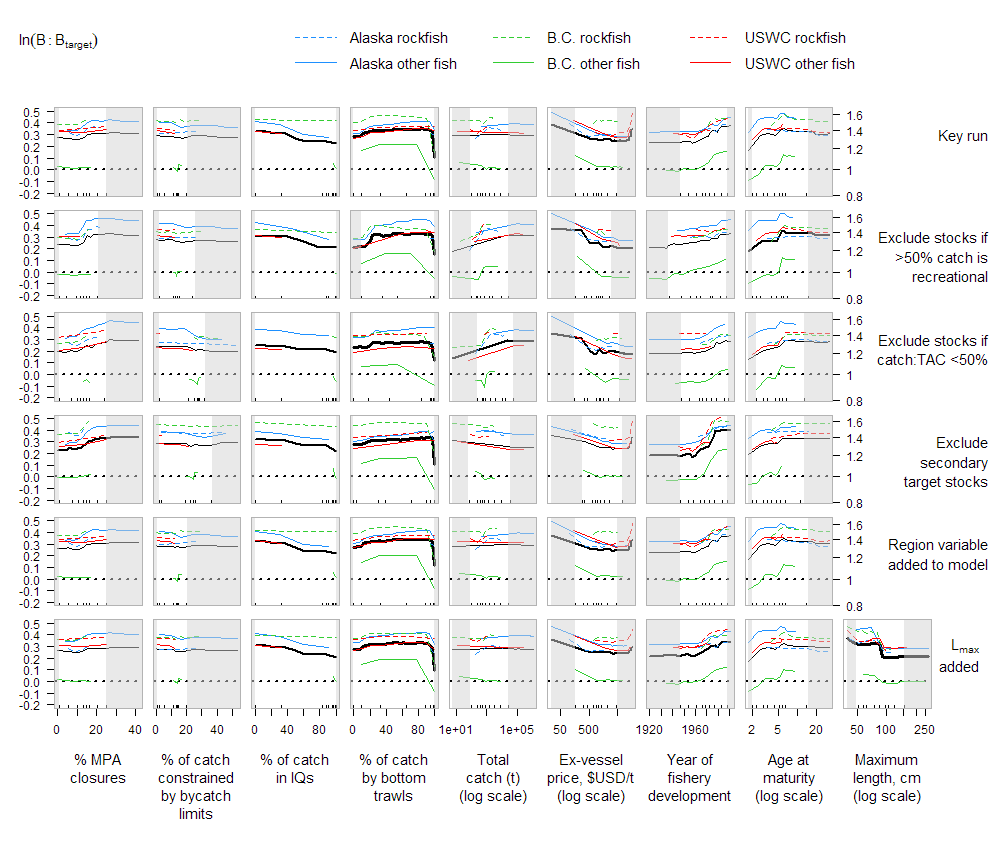

Supplement: Figure S13 — Partial dependence plots for sensitivity analyses showing the influence of numerical covariates on the mean B:Btarget ratio. The key run and five sensitivity scenarios are labelled in the right margin. See Fig. 3 caption in main text for further details. (TIF) [file pone.0056684.s013.tif]

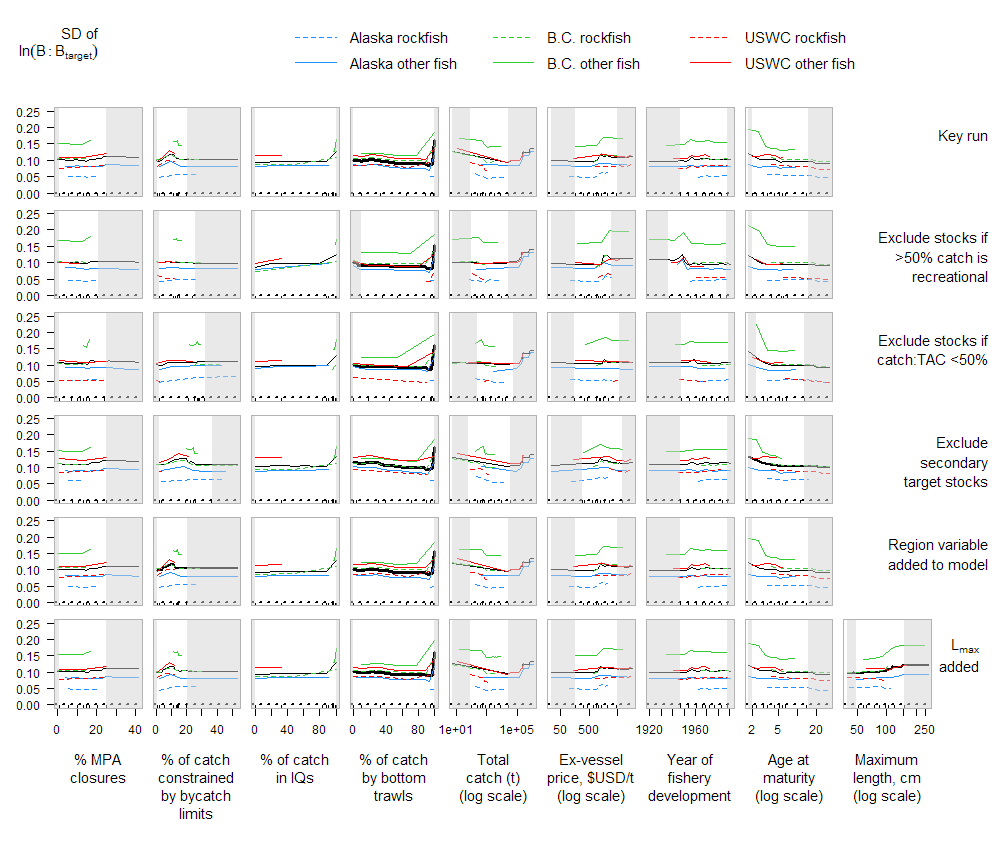

Supplement: Figure S14 — Partial dependence plots for sensitivity analyses showing the influence of numerical covariates on the interannual variability of the B:Btarget ratio. The key run and five sensitivity scenarios are labelled in the right margin. See Fig. 3 caption in main text for further details. (TIF) [file pone.0056684.s014.tif]

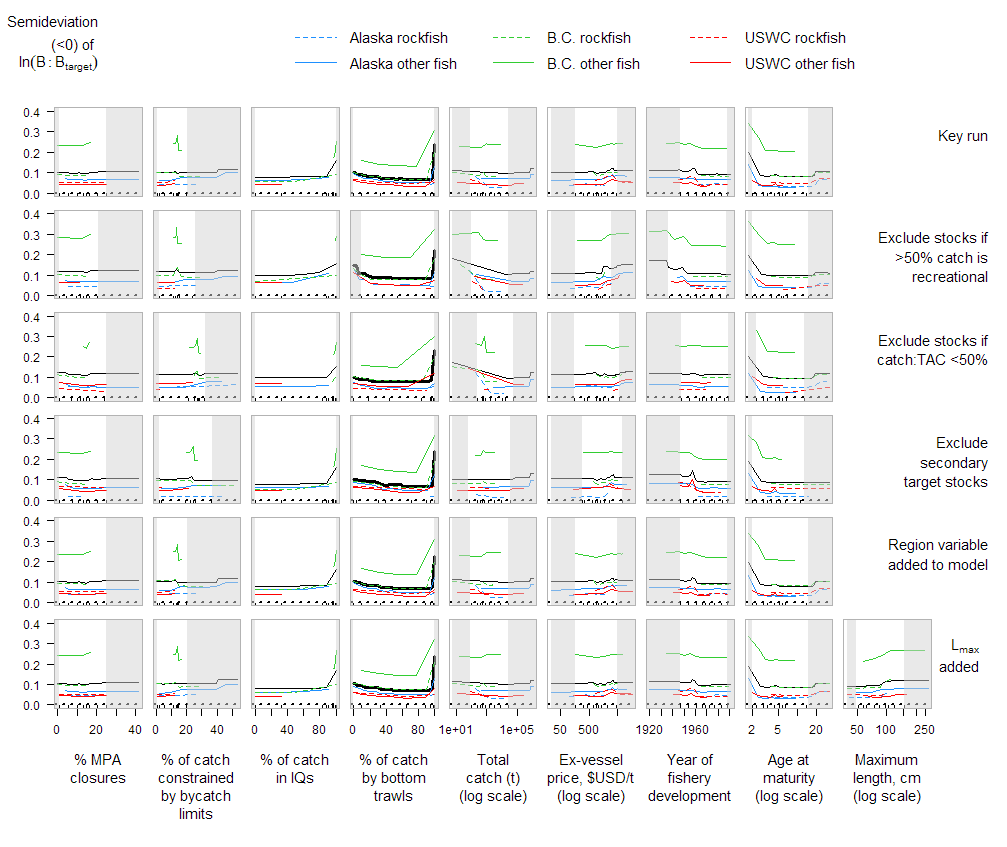

Supplement: Figure S15 — Partial dependence plots for sensitivity analyses showing the influence of numerical covariates on the semi-deviation of the B:Btarget ratio. The key run and five sensitivity scenarios are labelled in the right margin. See Fig. 3 caption in main text for further details. (TIF) [file pone.0056684.s015.tif]

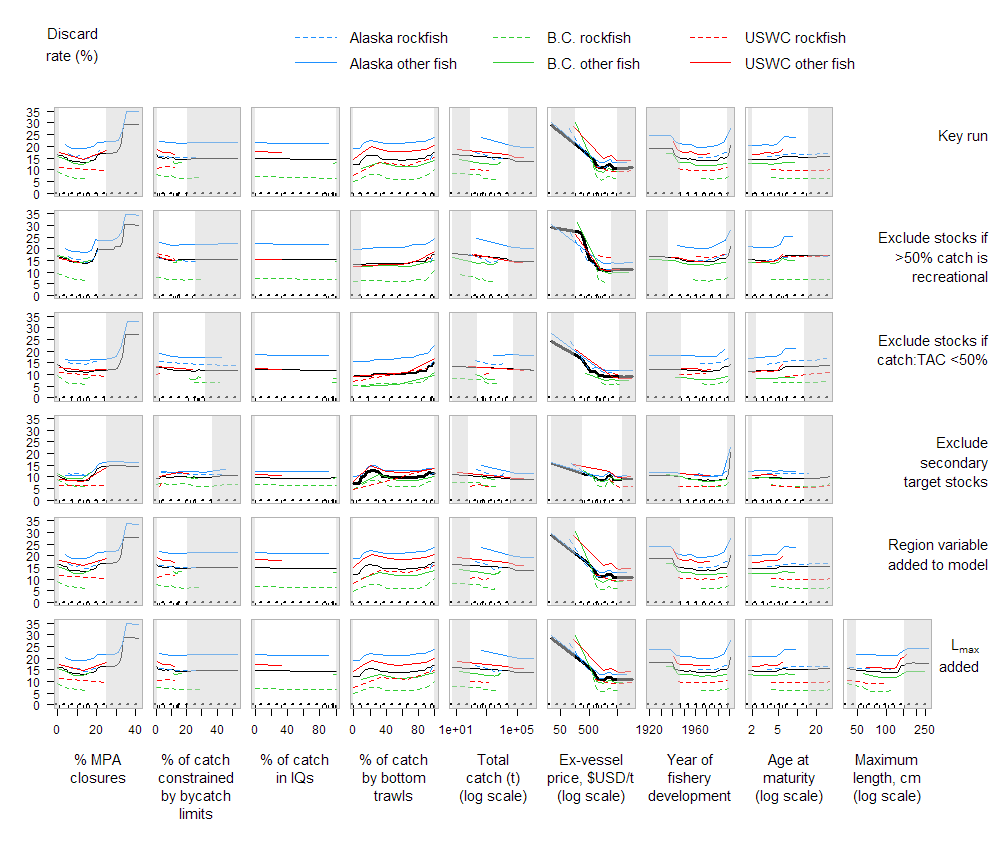

Supplement: Figure S16 — Partial dependence plots for sensitivity analyses showing the influence of numerical covariates on the proportion of catch discarded. The key run and five sensitivity scenarios are labelled in the right margin. See Fig. 3 caption in main text for further details. (TIF) [file pone.0056684.s016.tif]
