# Supplementary material for: Effects of Management Tactics on Meeting Conservation Objectives for Western North American Groundfish Fisheries
Source: PLoS One. 2013 Feb 27;8(2):e56684. doi: 10.1371/journal.pone.0056684 (PMC3584066; doi:10.1371/journal.pone.0056684)
Supplement: Table S1 — Stocks included in random forest data analyses (n = 85). (DOCX) [file pone.0056684.s017.docx]

**Table S1. Stocks included in random forest data analyses (n = 85).**

| **Region** | **Taxonomic and habitat association** | **Stock** | **Catch:TAC** | **F:F_target_** | **B:B_target_** | **Discard %** |
| --- | --- | --- | --- | --- | --- | --- |
| Alaska | Rockfish (demersal) | Bering Sea/Aleutian Is. northern rockfish | 1 | 1 | 1 | 1 |
|  |  | Bering Sea/Aleutian Is. Pacific ocean perch | 1 | 1 | 1 | 1 |
|  |  | Bering Sea/Aleutian Is. rougheye rockfish | 1 | 1 | 1 | 1 |
|  |  | Bering Sea/Aleutian Is. shortraker rockfish | 1 | 1 | 0 | 1 |
|  |  | Gulf of Alaska dusky rockfish | 1 | 1 | 1 | 1 |
|  |  | Gulf of Alaska northern rockfish | 1 | 1 | 1 | 1 |
|  |  | Gulf of Alaska Pacific ocean perch | 1 | 1 | 1 | 1 |
|  |  | Gulf of Alaska rougheye rockfish | 1 | 1 | 1 | 1 |
|  |  | Gulf of Alaska shortraker rockfish | 1 | 0 | 0 | 1 |
|  |  | Gulf of Alaska shortspine thornyhead | 1 | 0 | 0 | 1 |
|  | Rockfish (reef-associated) | Gulf of Alaska yelloweye rockfish | 1 | 0 | 0 | 1 |
|  | Other fish (demersal) | Bering Sea/Aleutian Is. Alaska plaice | 1 | 1 | 1 | 1 |
|  |  | Bering Sea/Aleutian Is. arrowtooth flounder | 1 | 1 | 1 | 1 |
|  |  | Bering Sea/Aleutian Is. atka mackerel | 1 | 1 | 1 | 1 |
|  |  | Bering Sea/Aleutian Is. flathead sole | 1 | 1 | 1 | 1 |
|  |  | Bering Sea/Aleutian Is. northern rock sole | 1 | 1 | 1 | 1 |
|  |  | Bering Sea/Aleutian Is. Pacific cod | 1 | 1 | 1 | 1 |
|  |  | Bering Sea/Aleutian Is. yellowfin sole | 1 | 1 | 1 | 1 |
|  |  | Gulf of Alaska arrowtooth flounder | 1 | 1 | 1 | 1 |
|  |  | Gulf of Alaska atka mackerel | 1 | 0 | 0 | 1 |
|  |  | Gulf of Alaska dover sole | 1 | 1 | 1 | 1 |
|  |  | Gulf of Alaska flathead sole | 1 | 1 | 1 | 1 |
|  |  | Gulf of Alaska Pacific cod | 1 | 1 | 1 | 1 |
|  |  | Gulf of Alaska rex sole | 1 | 1 | 1 | 1 |
|  |  | Pacific halibut (coastwide) | 1 | 1 | 1 | 1 |
|  |  | Sablefish | 1 | 1 | 1 | 1 |
|  | Other fish (benthopelagic) | Bering Sea/Aleutian Is. Greenland halibut | 1 | 1 | 1 | 1 |
|  |  | Aleutian Is. walleye pollock | 1 | 1 | 1 | 1 |
|  |  | Eastern Bering Sea walleye pollock | 1 | 1 | 1 | 1 |
|  |  | Gulf of Alaska walleye pollock | 1 | 1 | 1 | 1 |
| B.C. | Rockfish (demersal) | Longspine thornyheads | 1 | 0 | 0 | 1 |
|  |  | Pacific ocean perch | 1 | 1 | 1 | 1 |
|  |  | Redbanded rockfish | 0 | 0 | 0 | 1 |
|  |  | Redstripe rockfish | 1 | 0 | 0 | 1 |
|  |  | Rougheye rockfish | 1 | 0 | 0 | 1 |
|  |  | Sharpchin rockfish | 0 | 0 | 0 | 1 |
|  |  | Shortraker rockfish | 1 | 0 | 0 | 1 |
|  |  | Shortspine thornyheads | 1 | 0 | 0 | 1 |
|  |  | Silvergray rockfish | 1 | 0 | 0 | 1 |
|  |  | Yellowmouth rockfish | 1 | 1 | 1 | 1 |
|  | Rockfish (reef-associated) | Canary rockfish | 1 | 1 | 1 | 1 |
|  |  | Yelloweye rockfish | 1 | 0 | 0 | 1 |
|  |  | Yellowtail rockfish | 1 | 0 | 0 | 1 |
|  | Rockfish (pelagic) | Widow rockfish | 1 | 0 | 0 | 1 |
|  | Other fish (demersal) | Arrowtooth flounder | 0 | 0 | 0 | 1 |
|  |  | Big skate | 1 | 0 | 0 | 1 |
|  |  | Dover sole | 1 | 0 | 0 | 1 |
|  |  | Hecate Strait English sole | 1 | 1 | 1 | 1 |
|  |  | Hecate Strait Pacific cod | 1 | 1 | 1 | 1 |
|  |  | Hecate Strait rock sole | 1 | 1 | 1 | 1 |
|  |  | Longnose skate | 1 | 0 | 0 | 1 |
|  |  | Petrale sole | 1 | 1 | 1 | 1 |
|  |  | Sablefish | 1 | 1 | 1 | 1 |
|  |  | West Coast Vancouver Island English sole | 1 | 1 | 1 | 1 |
|  |  | West Coast Vancouver Island Pacific cod | 1 | 1 | 1 | 1 |
|  |  | West Coast Vancouver Island rock sole | 1 | 0 | 0 | 1 |
|  | Other fish (benthopelagic) | Lingcod 3C | 1 | 1 | 1 | 1 |
|  |  | Lingcod 3D | 1 | 1 | 1 | 1 |
|  |  | Lingcod 5AB | 1 | 1 | 1 | 1 |
|  |  | Lingcod 5CDE | 1 | 1 | 1 | 1 |
|  |  | Spiny dogfish inside | 1 | 0 | 0 | 1 |
|  |  | Spiny dogfish outside | 1 | 0 | 0 | 1 |
|  |  | Walleye pollock | 1 | 0 | 0 | 1 |
| USWC | Rockfish (demersal) | California scorpionfish (southern) | 1 | 1 | 1 | 0 |
|  |  | Gopher rockfish | 1 | 1 | 1 | 0 |
|  |  | Longspine thornyhead | 1 | 1 | 1 | 1 |
|  |  | Shortspine thornyhead | 1 | 1 | 1 | 1 |
|  |  | Splitnose rockfish | 1 | 1 | 1 | 0 |
|  | Rockfish (reef-associated) | Black rockfish (northern) | 1 | 1 | 1 | 1 |
|  |  | Black rockfish (southern) | 1 | 1 | 1 | 1 |
|  |  | Blue rockfish | 0 | 1 | 1 | 0 |
|  | Other fish (demersal) | Arrowtooth flounder | 1 | 1 | 1 | 1 |
|  |  | Cabezon (northern California) | 1 | 1 | 1 | 1 |
|  |  | Cabezon (southern California) | 0 | 1 | 1 | 1 |
|  |  | Dover sole | 1 | 1 | 1 | 1 |
|  |  | Kelp greenling | 0 | 1 | 1 | 1 |
|  |  | Lingcod (northern) | 1 | 1 | 1 | 1 |
|  |  | Lingcod (southern) | 0 | 1 | 1 | 1 |
|  |  | Longnose skate | 0 | 1 | 1 | 0 |
|  |  | Pacific cod | 1 | 0 | 0 | 0 |
|  |  | Sablefish | 1 | 1 | 1 | 1 |
|  |  | Starry flounder (northern) | 1 | 1 | 1 | 0 |
|  |  | Starry flounder (southern) | 0 | 1 | 1 | 0 |
|  | Other fish (benthopelagic) | Pacific whiting (coastwide) | 1 | 1 | 1 | 1 |
|  |  | Spiny dogfish | 1 | 1 | 1 | 1 |

The four data columns indicate whether response variable data are available (1) or not available (0) for each stock.
