# Supplementary material for: Effects of Management Tactics on Meeting Conservation Objectives for Western North American Groundfish Fisheries
Source: PLoS One. 2013 Feb 27;8(2):e56684. doi: 10.1371/journal.pone.0056684 (PMC3584066; doi:10.1371/journal.pone.0056684)
Supplement: Table S2 — Stocks excluded from stock status presentation and from random forest data analyses. (DOCX) [file pone.0056684.s018.docx]

**Table S2. Stocks excluded from stock status presentation and from random forest data analyses.**

| **Exclusion** | **Stock** | **Reason for exclusion** |
| --- | --- | --- |
| Included for stock status (Fig. 1) but excluded from random forest analyses | | |
|  | USWC bocaccio (southern) | Currently under rebuilding plan |
|  | USWC canary rockfish | Currently under rebuilding plan |
|  | USWC cowcod | Currently under rebuilding plan |
|  | USWC darkblotched rockfish | Currently under rebuilding plan |
|  | USWC Pacific ocean perch | Currently under rebuilding plan |
|  | USWC petrale sole | Currently under rebuilding plan |
|  | USWC widow rockfish | Currently under rebuilding plan |
|  | USWC yelloweye rockfish | Currently under rebuilding plan |
|  | USWC blackgill rockfish | Commonly co-caught with stocks under rebuilding |
|  | USWC chilipepper (southern) | Commonly co-caught with stocks under rebuilding |
|  | USWC English sole | Commonly co-caught with stocks under rebuilding |
|  | USWC yellowtail rockfish (northern) | Commonly co-caught with stocks under rebuilding |
| Excluded from both stock status (Fig. 1) and random forest analyses | | |
|  | B.C. bocaccio | No commercial value |
|  | USWC greenstriped rockfish | No commercial value |
|  | USWC shortbelly rockfish | No commercial value |
