# Supplementary material for: Effects of Management Tactics on Meeting Conservation Objectives for Western North American Groundfish Fisheries
Source: PLoS One. 2013 Feb 27;8(2):e56684. doi: 10.1371/journal.pone.0056684 (PMC3584066; doi:10.1371/journal.pone.0056684)
Supplement: Text S1 — This section contains the results of exploratory data analyses and sensitivity tests as mentioned in the main text. Also listed are the groundfish stocks that were included in analyses (Table S1), and stocks excluded from analyses (Table S2). (DOCX) [file pone.0056684.s019.docx]

# Supporting Information

This section contains the results of exploratory data analyses and sensitivity tests as mentioned in the main text. Also listed are the groundfish stocks that were included in analyses (Table S1), and stocks excluded from analyses (Table S2).

## Exploratory data analyses and model fits

Figure S1 shows mosaic plots of the three possible pair-wise combinations among the three categorical predictor variables. Strong departures from independence among predictor variables would be indicated by strongly disjointed horizontal lines in the first and second columns of each plot. We see only weak to moderate departures from independence between seasonal closures and harvest control rules with estimated reference points as well as between rockfish/non-rockfish and harvest control rules with estimated reference points.

Figure S2 shows all pair-wise combinations of the eight continuous predictor variables. Strong colinearity (i.e. departure from independence) among predictors would be shown by large positive or negative slopes in the co-plots and by large correlation coefficients. There was no evidence of strong colinearity among any of the predictor variables.

Figures S3–S5 show scatterplots of response variables vs. the eight continuous predictor variables. These figures show the raw data used in random forest analyses, and thus correspond to Figures 3–5 of the main text.

Figure S6 shows scatterplots of predicted vs. observed values for response variables, to visually assess model fit.

## Sensitivity tests

Five sensitivity analyses were conducted, listed in the *Methods* section. We repeated all random forest analyses after:

- excluding stocks that are predominantly caught in recreational fisheries
- excluding stocks with catch**:**TAC ratios <50%
- excluding stocks identified as being secondary targets
- adding a ‘region’ as a 3-level categorical predictor variable
- adding maximum length as a continuous predictor variable.

Partial dependence plots for these sensitivity analyses are shown in Figures S7–S16, with one figure for each response variable. Results for the key run, as presented in the main text, are shown for comparison. In general, there were few noteworthy changes as a result of filtering the dataset or adding an additional predictor variable.
